# Resting-state electroretinography reveals pathological retinal oscillations in retinitis pigmentosa mice and patients

**DOI:** 10.1038/s41467-026-75520-9

**Published:** 2026-07-10

**Authors:** David G. Litvin, Alexia Boizot, Andrea Corna, Aurélie Navarro, Josiane Keller, Marc O. Heuschkel, Adrien Roux, Günther Zeck, Hoai Viet Tran, Diego Ghezzi

**Affiliations:** 1 Ophthalmic and Neural Technologies Laboratory, Department of Ophthalmology, University of Lausanne, Hôpital ophtalmique Jules-Gonin, Fondation Asile des Aveugles, Lausanne, Switzerland; 2 Biomedical Electronics and Systems, Institute of Biomedical Electronics, Technische Universität Wien, Vienna, Austria; 3 Centre d’Investigation Clinique, Department of Ophthalmology, University of Lausanne, Hôpital ophtalmique Jules-Gonin, Fondation Asile des Aveugles, Lausanne, Switzerland; 4 Tissue Engineering Group, School of Engineering, Architecture and Landscape of Geneva (HEPIA), University of Applied Sciences and Arts Western Switzerland (HES-SO), Geneva, Switzerland; 5 Oculogenetics unit, Department of Ophthalmology, University of Lausanne, Hôpital ophtalmique Jules-Gonin, Fondation Asile des Aveugles, Lausanne, Switzerland; 6 Centre for Gene Therapy and Regenerative Medicine, King’s College London, London, UK

**Keywords:** Biomedical engineering, Neurophysiology, Retinal diseases

## Abstract

Retinal remodeling occurs in both retinitis pigmentosa and age-related macular degeneration. However, it is still unknown whether spared retinal circuits are also functionally altered. Functional changes have been observed in animal models of retinitis pigmentosa, including the emergence of bursting oscillatory activity in retinal ganglion cells. Yet, comparable oscillatory activity, or other functional alterations, has not been demonstrated in patients. To address this gap, here we report a non-invasive corneal neurotechnology measuring in-vivo resting-state electroretinography and analyzing its frequency content and temporal characteristics to identify biomarkers of functional remodeling. We document that retinal remodeling induces bursting oscillatory activity in-vivo in retinitis pigmentosa mouse models and translate these results to patients. Moreover, we showed in mice that bursting oscillatory activity can be pharmacologically modulated in-vivo. Furthermore, reducing this oscillatory activity increases retinal excitability to electrical stimulation. These results are crucial for a better understanding of retinal degeneration and contribute to sight restoration efforts.

## Abstract (web summary)

The authors present that a noninvasive corneal neurotechnology measuring in-vivo resting-state electrical activity of the retina detects biomarkers of functional retinal remodeling caused by degeneration.

## Introduction

Photoreceptor degeneration triggers a progressive anatomical remodeling of the retina^1,2^. First, photoreceptor stress initiates early remodeling and reprogramming events. Then, microglia, Müller glia and retinal pigmented epithelium cells become involved. The outer nuclear layer shrinks and cell stress pathways are engaged while Müller cells begin sealing the retina off from the choroid. Last, cell death, cell migration, dendrite retraction, and de-novo neurite formation lead to circuit rewiring, layer disruption and massive topological restructuring of the inner retina^3,4^. In contrast, amacrine cells^4^ and retinal ganglion cells (RGCs)^5–8^ partially survive and maintain their morphology.

In addition, animal models of retinitis pigmentosa (RP) revealed functional changes in retinal spontaneous activity during degeneration. First, hyperactivity of retinal origin has been identified in the primary visual cortex and the superior colliculus of retinal degeneration (RD) 1 mice^9^. Then, retinal explants from RD1 mice recorded with microelectrode arrays confirmed RGC bursting activity paired to an oscillatory activity (~8–15 Hz)^10^. Functional changes have been found in retinal explants from other models as well. Explanted retinas from RD10 mice presented a predominantly ~5 Hz bursting oscillatory activity^11,12^. N-methyl-N-nitrosourea (MNU) drug-induced retinal degeneration models also showed oscillatory activity ex-vivo at ~7 Hz in mice^13^ and ~20 Hz in Macaca fascicularis monkeys^14^. Experiments in mouse explanted retinas also showed that oscillatory activity is not intrinsic to RGCs and instead originates from synaptic inputs. However, several different sources are still proposed, including bipolar cells^15^, amacrine cells^12,16,17^, or electrically coupled bipolar cells and amacrine cells^18^. Furthermore, gap junctions are thought to allow fast propagation of oscillation across the retina^15,19,20^. Collectively, these findings indicate that RP-related retinal degeneration elicits robust model-dependent oscillatory activity that arise from altered synaptic inner-retinal circuitry.

While anatomical retinal remodeling has been described in human RP^21,22^, it remains unknown if spared retinal circuits in degenerating human retinas are also functionally altered. For example, there is no evidence of hyperactivity or oscillatory activity in human RP retinas. A fundamental limitation to understand functional changes in human retinas is the lack of methods to non-invasively measure spontaneous retinal activity in-vivo. Indeed, direct changes in retinal resting activity in-vivo have not yet been measured neither in animals nor in humans, despite earlier attempts in mice^23,24^. Nevertheless, monitoring resting retinal activity in-vivo could have important clinical and therapeutic consequences^22^. For example, bursting oscillatory activity acts as an intrinsic retinal pacemaker that makes the retina less responsive to external excitation^25,26^, increasing the stimulation threshold of RGCs^13,27–30^ and thereby reducing the efficacy of vision restoration approaches such as optogenetics and retinal implants.

To address this need, we propose a tailored flexible corneal neurotechnology and an analysis pipeline to measure resting-state electroretinography (rsERG) in-vivo, detect alterations in spontaneous retinal activity and find biomarkers indicating the presence of oscillatory activity in degenerating retinas.

## Results

### Resting-state ERG reveals oscillatory activity in end-stage RP RD1 mice in-vivo

**Fig. 1:**
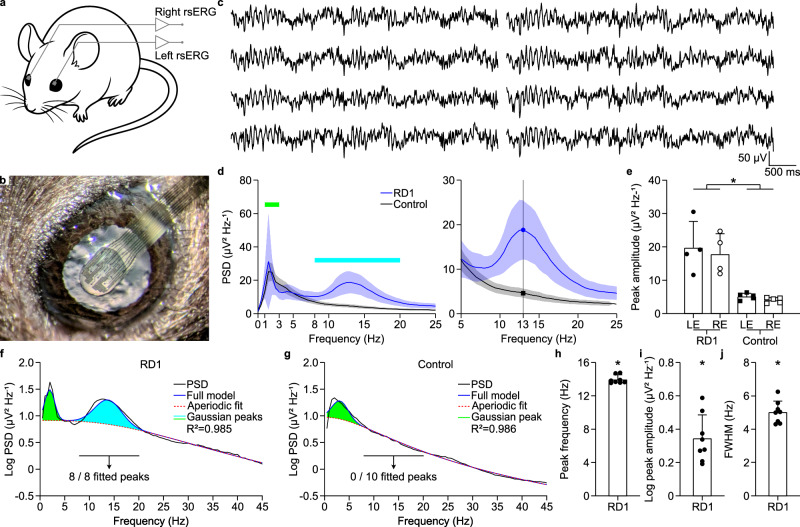
Pathology-specific frequency signature in end-stage RD1 retinas in-vivo. **a** Sketch of the experiment. Two flexible microelectrode arrays are placed onto both corneas in anesthetized mice. **b** Image of one flexible microelectrode array on the mouse cornea. **c** 5-s long band-pass filtered rsERG epochs recorded from the 8 microelectrodes in one RD1 mouse eye. **d** Mean eye average PSDs from RD1 mice in blue and control mice in black. The enlargement shows a magnification around the high-frequency PSD peak corresponding to bursting oscillatory activity. The shaded area indicates ± S.D. **e** Mean PSD peak amplitude at 13 Hz in RD1 and control mice (±S.D.). Sample size is *n* = 8 eyes from *N* = 4 mice (196-198 days old) for RD1 mice and *n* = 10 eyes from *N* = 5 mice (27 days old) for control mice. Full results of two-way ANOVA and Tukey’s multiple comparisons tests in Supplementary Data 1. RD1 vs Control: * p < 0.0001. **f**, **g** Fitting process in eye average PSDs from one RD1 eye (**f**) and one control eye (**g**). In black is the original eye average PSD, in blue the full model fit, in red the aperiodic component and in green/cyan the Gaussian peaks. **h–j** Mean peak frequency (**h**), mean log peak amplitude (**i**) and mean FWHM (**j**) of the fitted high-frequency peaks in RD1 eye average PSDs (±S.D.). Sample size is *n* = 8 eyes from *N* = 4 mice (196–198 days old) for RD1 mice and *n* = 10 eyes from *N* = 5 mice (27 days old) for control mice. Two-sided one sample *t*-test for peak frequency: * *p* < 0.0001, t(7) = 79.79, 95% CI [13.63, 14.46] and Cohen’s d = 28.22 (**h**). Two-sided one sample *t*-test for peak amplitude: * *p* = 0.0002, t(7) = 6.92, 95% CI [0.23, 0.47] and Cohen’s d = 2.45 (**i**). Two-sided one sample t-test for FWHM: * *p* < 0.0001, t(7) = 21.27, 95% CI [4.49, 5.61] and Cohen’s d = 7.52 (**j**). Source data are provided as a Source Data file.

First, we assessed the presence of bursting oscillatory activity in-vivo in four end-stage 28-weeks old RD1 mice and compared results to five sighted 4-weeks old C57BL/6 J control mice. We measured rsERG using a non-invasive flexible corneal neurotechnology with local grounding (8 microelectrodes plus a local large ground electrode in each array)^31^ positioned on the cornea of both left (LE) and right (RE) eyes (Fig. 1a, b). The advantages of a flexible microelectrode array with local grounding are several: (i) the thin-film array tightly adheres to the cornea, providing a higher signal-to-noise ratio; (ii) the flexible strip design dampens small head movements reducing movement artifacts on the recorded rsERG; (iii) local grounding reduces baseline fluctuations due to eye movements (eye dipole rotation); (iv) the array of microelectrodes provide multiple signal sources improving signal processing.

Recordings lasted for 60 min, corresponding to the typical time at which mice began to awaken from general anesthesia. Signals were initially analyzed in the spectral domain. We segmented 60-min long data from every microelectrode of the array into 5-s epochs, subsequently band-pass filtered from 1 to 45 Hz (Fig. 1c). Next, we computed the power spectral density (PSD) for each epoch, performed artifact rejection, and averaged them into a single PSD for each microelectrode, here called ‘electrode average PSD’ (Supplementary Fig. 1). Last, we averaged together the eight electrode average PSDs from each microelectrode into a single PSD, here called ‘eye average PSD’. The eye average PSDs from rsERG recordings in RD1 mice display two frequency peaks (blue in Fig. 1d): one in the low-frequency range (1–3 Hz, green bar) and the other in the high-frequency range (8–20 Hz, cyan bar). The low-frequency peak, also visible in control mice (black in Fig. 1d), presumably corresponds to artifacts induced by physiological breathing, which is about 150 breaths per min (2.5 Hz) under ketamine/xylazine anesthesia^32^. In contrast, the high-frequency peak, coherent with the presence of bursting oscillatory activity^9,15^, is visible in RD1 mice but not in control mice. In particular, at the 13-Hz peak (Fig. 1d, enlargement), the eye average PSD power is significantly higher in RD1 mice compared to control mice (Fig. 1e). Here, we treated each eye as an independent statistical unit even if they originate from the same animal. Nevertheless, to rule out pseudoreplication, a two-way ANOVA test indicates that significance is only related to the difference between RD1 and controls regardless of the tested eyes (results of two-way ANOVA and Tukey’s multiple comparisons tests in Supplementary Data 1).

It should be noted that the peak shape in the low-frequency range is largely created by the band-pass filter low cutoff frequency (at 1 Hz) which removes the DC offset from the slow component of the signal, leaving the power component related to the breathing artifact (Supplementary Fig. 2a). Ultimately, this low-frequency artifact is a combination of mechanical movement due to physiological breathing, filter settings and the artifact rejection process. Therefore, it has a variable amplitude across microelectrodes in the same array, across time in the same eye, between the two eyes of a mouse, and across mice. In contrast, the amplitude of the oscillatory high-frequency peak remains stable across microelectrodes in the same array (Supplementary Fig. 2b, c). The coefficient of variation for the low-frequency peak is significantly higher than the one for the high-frequency peak (result of one-sided paired *t*-test in Supplementary Fig. 2c). Importantly, we verified that the amplitude of the high-frequency PSD peak is not correlated to the amplitude of the low-frequency breathing-related artifact (Spearman’s correlation coefficient *r* = -0.18, *p* = 0.0061; Supplementary Fig. 2d), indicating that variability in the breathing rate does not alter oscillatory activity.

Next, to quantitatively compare oscillatory activity across mice and further reduce the dependency of oscillatory activity (high-frequency PSD peak) from the aperiodic signal (including the breathing-related artifact), the eye average PSDs (black in Fig. 1f, g) underwent a Gaussian fitting process (Fitting Oscillations & One-Over-F, FOOOF), to identify oscillatory PSD peaks (green and cyan in Fig. 1f, g) after removing the aperiodic (or non-oscillatory) component (red in Fig. 1f, g) from the full model fit (blue in Fig. 1e, f)^33^. Parameters were optimized to best fit the high-frequency peak corresponding to oscillatory activity (cyan in Fig. 1f, g) and not the low-frequency artifact (green in Fig. 1f, g). Across RD1 eyes, the mean goodness of fit is R^2^ = 0.98 ± 0.02 while it is R^2^ = 0.98 ± 0.01 across control mice (±S.D.). The low-frequency peak corresponding to the breathing-induced artifact is fitted in all RD1 and control eye average PSDs (green in Fig. 1f, g). However, we discarded these data from further analysis since the peak is artificially created by the filter and it is not linked to neuronal oscillatory activity. The high-frequency oscillatory peak is fitted in all RD1 eye average PSDs (cyan in Fig. 1f) but not in control eye average PSDs (Fig. 1g). This fitted peak in RD1 mice has a mean peak frequency of 14.05 ± 0.50 Hz (± S.D.; Fig. 1h), mean log peak amplitude of 0.35 ± 0.14 µV^2^ Hz^-1^ ( ± S.D.; Fig. 1i), and mean full width at half maximum (FWHM) of 5.05 ± 0.67 Hz (± S.D.; Fig. 1j). All parameters are significantly higher than zero (results of two-sided one sample t-tests in Fig. 1h–j).

This first dataset demonstrates that non-invasive corneal rsERG can detect in-vivo changes in resting retinal activity such as the oscillatory activity emerging in RGCs after retinal remodeling. The dominant frequency observed in this dataset (~13–15 Hz) is slightly higher than the one previously reported from ex-vivo recordings in retinal explants (~9–10 Hz)^11,15,18^. We hypothesize that this difference is due to the age of the mice in this dataset and the corresponding progression of the disease (end-stage). This hypothesis and the age difference between sighted controls and RD1 mice in this dataset is addressed in the next section (Supplementary Fig. 3).

### Oscillatory activity in RD1 mice is visible from its onset

Prior ex-vivo experiments in RD1 explanted retinas showed that bursting oscillatory activity begins at postnatal week (PNW) 4 in some RGCs and reaches its maximum at PNW 8^34^. Hence, we tested whether in-vivo rsERG could also reveal oscillatory activity as early as PNW 4.

**Fig. 2:**
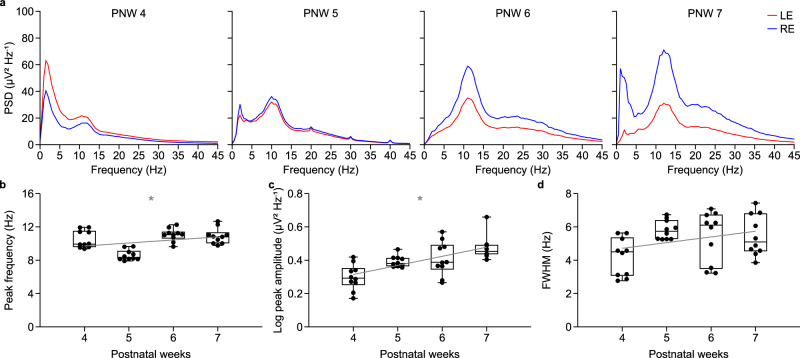
Onset detection of oscillatory activity. **a** Eye average PSDs from one RD1 mouse at PNW 4, PNW 5, PNW 6 and PNW 7 (respectively 29, 36, 41 and 47 days old). **b–d** Peak frequency (**b**), log peak amplitude (**c**), and FWHM (**d**) of the fitted Gaussian peaks corresponding to the bursting oscillatory activity across weeks. Boxes extend from the 25^th^ to 75^th^ percentiles, whiskers extend from the min to max values, and the line is the median value. Sample size is *n* = 10 eyes from *N* = 5 mice at each PNW. The gray lines are the linear regression. For peak frequency: R^2^ = 0.11; F-test: * *p* = 0.0432, F(1,38) = 4.376, 95% CI [0.01 to 0.73] and Cohen’s f^2^ = 0.12 (**b**). For log peak amplitude: R^2^ = 0.42; F-test: * *p* < 0.0001, F(1,38) = 26.86, 95% CI [0.03, 0.08] and Cohen’s f^2^ = 0.71 (**c**). For FWHM: R^2^ = 0.10; F-test: *p* = 0.0585, F(1,38) = 3.805, 95% CI [-0.01, 0.70] and Cohen’s f^2^ = 0.10 (**d**). Source data are provided as a Source Data file.

We recorded five RD1 mice for 60 min at PNW 4 (29 days old), PNW 5 (36 days old), PNW 6 (41 days old), and PNW 7 (47 days old). An oscillatory peak in the eye average PSD is detected already at PNW 4. Furthermore, PSD peaks become more robust over the weeks (Fig. 2a). At PNW 4, the eye average PSD power at peak (10.5 Hz) in RD1 mice is significantly higher than aged-matched controls regardless of the tested eyes (results of ANOVA and Tukey’s multiple comparisons tests in Supplementary Fig. 3).

The PSD peaks in RD1 mice could be Gaussian fitted in all tested eyes, at all tested PNWs. The mean goodness of fit is R^2^ = 0.98 ± 0.01 at PNW 4, R^2^ = 0.97 ± 0.01 at PNW 5, R^2^ = 0.89 ± 0.06 at PNW 6, and R^2^ = 0.91 ± 0.01 at PNW 7 (±S.D.). Peak frequency, log peak amplitude, and FWHM have been interpolated with a linear regression showing increase over the weeks (gray lines in Fig. 2b–d). The fitted slopes are significantly non-zero for log peak amplitude (results of F-test in Fig. 2b) and for peak frequency (results of F-test in Fig. 2c). The fitted slope for FWHM is slightly not significantly non-zero (results of F-test in Fig. 2d).

This dataset demonstrates that rsERG is sufficiently robust to detect oscillatory activity already at its onset at PNW 4 (the earliest time point at which rhythmic oscillations have been reported in ex-vivo explanted RD1 retinas) and to monitor its progression through the weeks while retinal degeneration worsens. Also, younger RD1 mice display oscillatory activity at a dominant frequency (~8–12 Hz) closer to previous reports on ex-vivo recordings in retinal explants (~9–10 Hz)^11,15,18^.

### Oscillatory activity in RD1 mice is modulated pharmacologically

Next, we investigated whether oscillatory activity can be modulated in-vivo by pharmacological intervention. Specifically, the combination of glycinergic and GABA_A_ receptor blockers was shown ex-vivo to increase amplitude and synchronization of neuronal oscillations in both RD1 and RD10 explanted retinas^15,35^. Given this dependence of oscillatory activity on GABA_A_ receptors, we tested whether the systemic administration of diazepam, a positive allosteric modulator of GABA_A_ receptors, could suppress retinal oscillatory activity in-vivo.

**Fig. 3:**
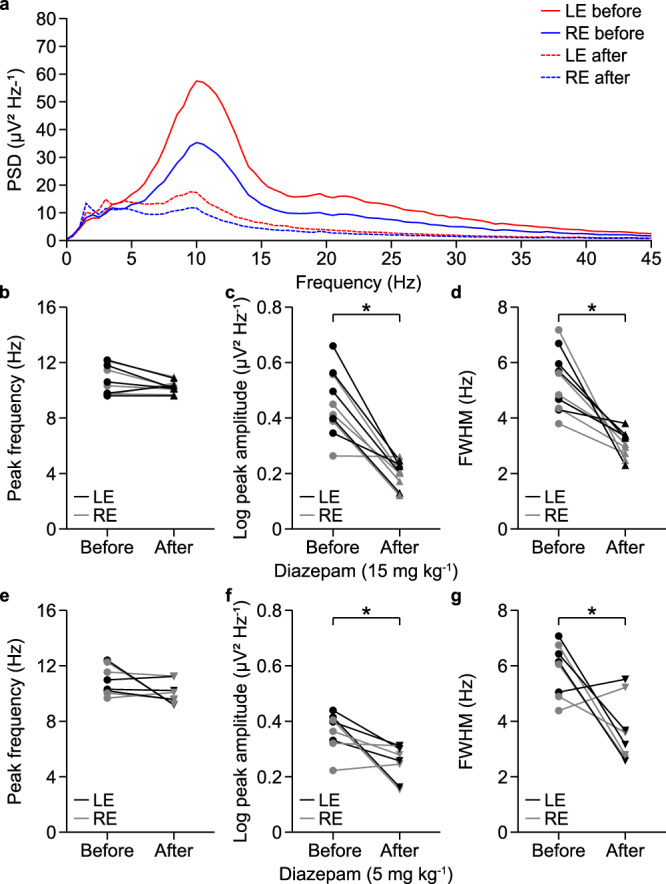
Pharmacological manipulation in-vivo of oscillatory activity. **a** Eye average PSDs from one RD1 mouse before (solid lines) and after (dashed lines) diazepam administration at 15 mg kg^-1^. **b–d** Peak frequency (**b**), log peak amplitude (**c**), and FWHM (**d**) of the fitted Gaussian peaks before and after administration of diazepam at 15 mg kg^-1^. Sample size is *n* = 8 eyes from *N* = 4 mice (56–59 days old). Full results of two-way repeated measures ANOVA and Šídák’s multiple comparisons tests in Supplementary Data 1. Before vs after: *p* = 0.1277 for peak frequency (**b**), * *p* = 0.0002 for log peak amplitude (**c**), and * *p* = 0.0014 for FWHM (**d**). **e–g** Peak frequency (**e**), log peak amplitude (**f**), and FWHM (**g**) of the fitted Gaussian peaks before and after administration of diazepam at 5 mg kg^-1^. Sample size is *n* = 10 eyes from *N* = 5 mice (52–55 days old). Full results of two-way repeated measures ANOVA and Šídák’s multiple comparisons tests in Supplementary Data 1. Before vs after: *p* = 0.1566 for peak frequency (**e**), * *p* = 0.0250 for log peak amplitude (**f**), and * *p* = 0.0248 for FWHM (**g**). Source data are provided as a Source Data file.

We measured rsERG in five RD1 mice (56–59 days old) for 30 min before and 30 min after intraperitoneal administration of diazepam (15 mg kg^-1^) with a 10 min gap between recordings to let diazepam take effect. In all eyes, the oscillatory peak in the eye average PSD is nearly abolished by diazepam (Fig. 3a). Diazepam significantly reduced the mean log peak amplitude from 0.45 ± 0.12 to 0.20 ± 0.05 µV^2^ Hz^-1^ and the mean FWHM from 5.31 ± 0.10 to 3.07 ± 0.46 Hz with treatment being the only significant effect (± S.D.; Fig. 3c, d; results of two-way repeated measures ANOVA and Šídák’s multiple comparisons tests in Supplementary Data 1). In contrast, the mean peak frequency remained unchanged from 10.74 ± 1.06 to 10.26 ± 0.44 Hz (±S.D.; Fig. 3b; results of two-way repeated measures ANOVA in Supplementary Data 1). The mean goodness of fit is R^2^ = 0.93 ± 0.04 before and R^2^ = 0.99 ± 0.01 after diazepam (±S.D.). To rule out the possibility that the reduction of the high-frequency oscillatory peak is related to a systemic action of diazepam and not to a specific retinal effect (for example a change in depth of anesthesia and physiological breathing), we verified that the decrease in the high-frequency PSD peak power is not correlated with changes in the low-frequency breathing-related artifact (Supplementary Fig. 4). This result indicates that the reduction in oscillatory activity is unlikely to be driven by systemic physiological effects of diazepam.

Then, we repeated the experiment in four additional RD1 mice (52–55 days old) with a lower dose of diazepam (5 mg kg^-1^) yielding the same results but with a lower effect (Fig. 3e-g). Diazepam significantly reduced the mean log peak amplitude from 0.36 ± 0.07 to 0.25 ± 0.06 µV^2^ Hz^-1^ and the mean FWHM from 5.86 ± 0.96 to 3.65 ± 1.14 Hz with treatment being the only significant effect (± S.D.; results of two-way repeated measures ANOVA and Šídák’s multiple comparisons test in Supplementary Data 1). On the contrary, the mean peak frequency remained unchanged from 10.94 ± 1.05 to 10.06 ± 0.82 Hz (±S.D.; results of two-way repeated measures ANOVA in Supplementary Data 1). The mean goodness of fit is R^2^ = 0.95 ± 0.02 before and R^2^ = 0.99 ± 0.01 after diazepam (± S.D.).

Finally, prior ex-vivo experiments in RD1 explanted retinas provided contrasting evidence on the role of voltage-gated sodium channels in maintaining oscillatory activity. One study showed that blocking voltage-gated sodium channels with tetrodotoxin abolishes RGC spiking activity without reducing neuronal oscillations^15^, while another reported that both spiking and oscillatory activities are reduced in RGCs^19^. A possible explanation for this discrepancy is the different dose used in the two studies: a lower dose (0.2 µM) in the first one and a higher dose in the second one (1 µM)^19^. Oxybuprocaine is a local anesthetic binding voltage-gated sodium channels routinely used in ophthalmology, including during light-evoked ERG. Therefore, we tested whether the topical administration of oxybuprocaine (0.4% eye drops) alters in-vivo rsERG. We recorded rsERG in five RD1 mice (49–51 days old) for 30 min before and 30 min after topical administration in one eye only, with a 10 min gap between recordings, using the untreated eye as an internal control (Supplementary Fig. 5). Peak frequency and log peak amplitude remained unchanged in both the treated and untreated eyes. Only, the FWHM showed a slight decrease in both groups (results of two-way repeated measures ANOVA and Šídák’s multiple comparisons tests in Supplementary Fig. 5). The mean goodness of fit is R^2^ = 0.99 ± 0.01 before, R^2^ = 0.99 ± 0.01 in the untreated eyes and R^2^ = 0.99 ± 0.01 in the treated eyes (±S.D.).

Although the systemic administration of diazepam precludes definitive attribution of the observed effects specifically to retinal GABA_A_ receptors and does not fully exclude extra-retinal contributions, these data together with previous ex-vivo findings demonstrate that retinal oscillatory activity is markedly suppressed in-vivo by a positive allosteric modulator of GABA_A_ receptors. This result has translational relevance, as previous studies in explanted mouse retinas have shown that suppressing bursting oscillatory activity improves the efficacy of both electrical and optogenetic retinal stimulation^25,36^. In addition, the commonly used local anesthetic oxybuprocaine did not alter rsERG detection capability. Since oxybuprocaine eye drops are not designed to reach the retina at a therapeutically meaningful concentration, this result is consistent with the previous ex-vivo observation in RD1 explanted retinas showing that a low dose of tetrodotoxin does not diminish retinal oscillatory activity^15^. This result further supports the clinical translatability of rsERG since oxybuprocaine is frequently used in ERG recordings with corneal lens electrodes.

### Retinal stimulation efficacy increases by blocking oscillatory activity

Previous ex-vivo studies have shown that bursting oscillatory activity acts as an intrinsic retinal pacemaker, reducing retinal responsiveness to external excitation^25,26^ by increasing the stimulation threshold of RGCs^13,27–30^. Based on this evidence, we hypothesized that blocking oscillatory activity in-vivo could enhance the efficacy of retinal stimulation.

**Fig. 4:**
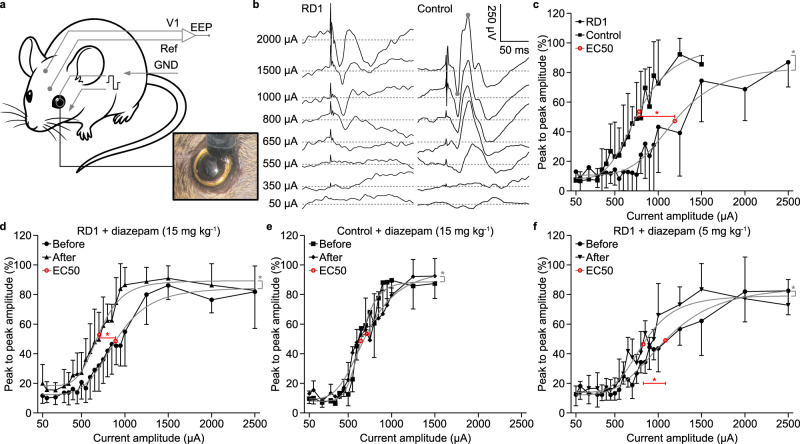
Reducing periodic bursting activity increased retinal excitability. **a** Sketch of the experiment. A ERG-jet electrode is placed on the eye for TcES. A needle electrode is inserted underneath the eye as a return electrode. EEPs are recorded from the contralateral V1. **b** Examples of EEPs in RD1 (left) and control (right) mice at increasing current intensities. The gray dots indicate the first negative (N1) and first positive (P1) peaks used to quantify the response amplitude. **c** Mean normalized input-output response curves for RD1 mice (±S.D.; *n* = 9 eyes from *N* = 5 mice, 62–72 days old) and control mice (±S.D.; *n* = 10 eyes from *N* = 5 mice, 57–70 days old). One RD1 mouse died before we could finish the experiment in the second eye. For sigmoidal fits: F-test, F(4,390) = 39.06, * *p* < 0.0001. For EC50: F-test: F(1390) = 8.66, * *p* = 0.0031. **d** Mean normalized input-output response curves for RD1 mice (±S.D.; *n* = 5 eyes from *N* = 5 mice, 55–57 days old) before and after diazepam administration at 15 mg kg^-1^. For sigmoidal fits: F-test, F(4,212) = 20.78, * *p* < 0.0001. For EC50: F-test, F(1,212) = 16.70, * *p* < 0.0001. **e** Mean normalized input-output r**e**sponse curves for control mice (±S.D.; *n* = 3 eyes from *N* = 3 mice, 55 days old) before and after diazepam administration at 15 mg kg^-1^. For sigmoidal fits: F-test, F(4,112) = 4.052, * *p* = 0.0042. For EC50: F-test, F(1,112) = 2.51, *p* = 0.1159. **f** Mean normalized input-output response curves for RD1 mice (±S.D.; *n* = 5 eyes from *N* = 5 mice, 55–59 days old) before and after diazepam administration at 5 mg kg^-1^. For sigmoidal fits: F-test, F(4212) = 8.04, * *p* < 0.0001. For EC50: F-test, F(1212) = 13.03, * *p* = 0.0004. In (**c–f**), the gray lines are the four parameter logistic regressions. Torus rings indicate the EC50 points. The red bars indicate a significant change in the EC50 value. Source data are provided as a Source Data file.

To test this hypothesis, we assessed retinal excitability upon transcorneal electrical stimulation (TcES) delivered through an ERG-jet electrode while recording electrically evoked potentials (EEPs) from the primary visual cortex (V1) contralateral to the stimulated eye (Fig. 4a). EEPs in RD1 mice (62–72 days old) have markedly lower peak-to-peak amplitude in comparison to EEPs in control mice (57–70 days old), indicating reduced retinal excitability (Fig. 4b and Supplementary Fig. 6a). For this reason, additional higher current levels were used in RD1 mice and not in controls (2000 and 2500 µA). To control for non-retinal experimental biases such as potential difference in cortical responsivity between RD1 and control mice, responses were normalized, prior to curve fitting and EC50 estimation. Sigmoidal fits of the normalized responses differ significantly between RD1 and control mice (Fig. 4c). In addition, control mice exhibit a significantly lower EC50 current amplitude compared to RD1 mice (778.3 µA for control mice and 1191 µA for RD1 mice; Fig. 4c). One RD1 mouse died before completion of the experiment in the second eye.

Building on this result, in a second cohort of five RD1 mice (55-57 days old), we compared EEPs elicited by TcES in one eye before and after intraperitoneal injection of diazepam (15 mg kg^-1^). In absolute terms, EEPs exhibit lower peak-to-peak amplitude following diazepam administration (Supplementary Fig. 6b), consistent with systemic dosing affecting both brain-wide physiology and retinal activity. Accordingly, we also observed lower EEP amplitudes in three control mice (55 days old) following intraperitoneal injection of diazepam (15 mg kg^-1^; Supplementary Fig. 6c). To control for extra-retinal effects of diazepam, we again normalized responses, prior to curve fitting and EC50 estimation. In RD1 mice, sigmoidal fits differ significantly before and after diazepam administration (Fig. 4d) and diazepam significantly reduced the EC50 from 891.8 to 704.3 µA (Fig. 4d). In contrast, in control mice, although sigmoidal fits also differ significantly (Fig. 4e), the EC50 is not significantly changed following diazepam administration (643.6 µA before versus 713.8 µA after injection; Fig. 4e) further supporting a retina-specific effect. We observed a similar effect using a lower diazepam dose (5 mg kg^-1^) in five RD1 mice (55–59 days old). Normalized sigmoidal fits differ significantly before and after treatment (Fig. 4f), and the EC50 is significantly reduced following diazepam injection (Fig. 4f). Absolute input-output responses are shown in Supplementary Fig. 6d.

In summary, this dataset demonstrates that the impact of retinal oscillatory activity is not limited to reduced retinal responsiveness but also propagates downstream by impairing the retina ability to effectively drive V1 neurons. Crucially, when retinal oscillatory activity is reduced or suppressed in-vivo using a GABA_A_ receptor positive allosteric modulator, retinal excitability to exogenous stimulation is enhanced together with its output to V1 neurons. These findings suggest that preventing the emergence of oscillatory activity or suppressing it might represent a critical step for vision restoration strategies in end-stage RP patients, including visual prostheses, optogenetics or even regenerative approaches.

### Oscillatory activity in-vivo is present across RP mouse models

Having established that rsERG can detect oscillatory activity in RD1 mice, we next examined whether similar activity could also be detected in other RP mouse models.

First, we focused on RD10 mice, as previous ex-vivo studies in retinal explants have identified a predominant ~5 Hz bursting oscillatory activity beginning at PNW 8^11,35,37^. Notably, one study also showed that diazepam suppressed bursting oscillatory activity ex-vivo in RD10 explanted retinas^25^. However, detecting a ~5 Hz oscillatory activity in-vivo is challenging because its spectral peak can be masked by the 1–3 Hz breathing artifact. To address this issue, we modified the recording protocol by suppressing spontaneous breathing with a paralytic agent and mechanically ventilating the mice at 60 breath per minute (bpm) using 100% oxygen to prevent hypoxia. This technically demanding preparation reduced the breathing-related artifact to ~1 Hz, thereby improving the ability to resolve neuronal oscillations at ~5 Hz. Because mechanical ventilation requires a stable and long-lasting depth of anesthesia, ketamine/xylazine was replaced with urethane, which provides sustained anesthesia without the fluctuations associated with repeated ketamine/xylazine dosing. Importantly, rhythmic hyperactivity in the superior colliculus and visual cortex in RD1 mice under urethane anesthesia was already reported^9^, supporting the suitability of urethane for this experiment.

**Fig. 5:**
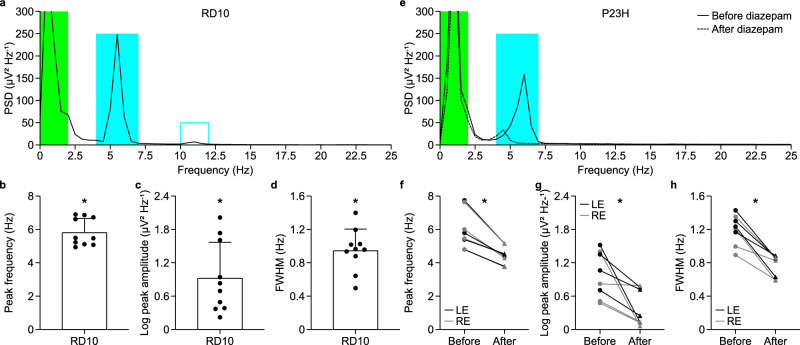
Periodic bursting activity in RD10 and P23H models. **a** Eye average PSD from a RD10 mouse eye. **b–d** Peak frequency (**b**), log peak amplitude (**c**) and FWHM (**d**) of the fitted Gaussian peaks corresponding to the periodic bursting activity (mean ± S.D.). Sample size is *n* = 10 eyes from *N* = 5 mice (101-260 days old). Two-sided one sample *t*-test for peak frequency: * p < 0.0001, t(9) = 22.46, 95% CI [5.30, 6.49] and Cohen’s d = 7.10 (**b**). Two-sided one sample t-test for log peak amplitude: * *p* = 0.0013, t(9) = 4.61, 95% CI [0.48, 1.40] and Cohen’s d = 1.46 (**c**). Two-sided one sample t-test for FWHM: * *p* < 0.0001, t(9) = 11.88, 95% CI [0.78, 1.14] and Cohen’s d = 3.76 (**d**). **e** Eye average PSD from a P23H mouse eye before and after diazepam at 15 mg kg^-1^. **f–h** Peak frequency (**f**), log peak amplitude (**g**) and FWHM (**h**) of the fitted Gaussian peaks corresponding to the periodic bursting activity before and after before and after administration of diazepam at 15 mg kg^-1^. Sample size is *n* = 8 eyes from *N* = 4 mice (140–146 days old). Two-sided one sample *t*-test for peak frequency before: * p < 0.0001, t(7) = 14.58, 95% CI [4.99, 6.92] and Cohen’s d = 5.15 (**f**). Two-sided one sample t-test **f**or log peak amplitude before: * *p* = 0.0003, t(7) = 6.74, 95% CI [0.64, 1.33] and Cohen’s d = 2.38 (**g**). Two-sided one sample t-test for FWHM before: * *p* < 0.0001, t(7) = 18.96, 95% CI [1.05, 1.35] and Cohen’s d = 6.70 (**h**). Full results of two-way repeated measures ANOVA and Šídák’s multiple comparisons tests in Supplementary Data 1. Before vs after: * *p* = 0.0014 for peak frequency (**f**), * *p* = 0.0163 for log peak amplitude (**g**), and * *p* = 0.0004 for FWHM (**h**). Source data are provided as a Source Data file.

We recorded rsERG in five RD10 mice (101–102, 117–120, and 260 days old). The eye average PSD shows a sharp peak at ~1 Hz (green rectangle in Fig. 5a) corresponding to mechanical ventilation followed by a second peak at ~5 Hz (cyan rectangle in Fig. 5a) consistent with the known frequency for bursting oscillatory activity in RD10 mice. A second harmonic at twice the fundamental frequency (~11 Hz) was also observed, reflecting the non-sinusoidal nature of neuronal oscillations (cyan box in Fig. 5a). Following fitting, the oscillatory peak exhibited a mean peak frequency of 5.89 ± 0.83 Hz (±S.D.; Fig. 5b), mean log peak amplitude of 0.94 ± 0.64 µV^2^ Hz^-1^ ( ± S.D.; Fig. 5c), and mean FWHM of 0.96 ± 0.26 Hz (±S.D.; Fig. 5d). All parameters are significantly higher than zero (results of two-sided one sample t-tests in Fig. 5b–d). The mean goodness of fit is R^2^ = 0.98 ± 0.02 ( ± S.D.).

**Fig. 6:**
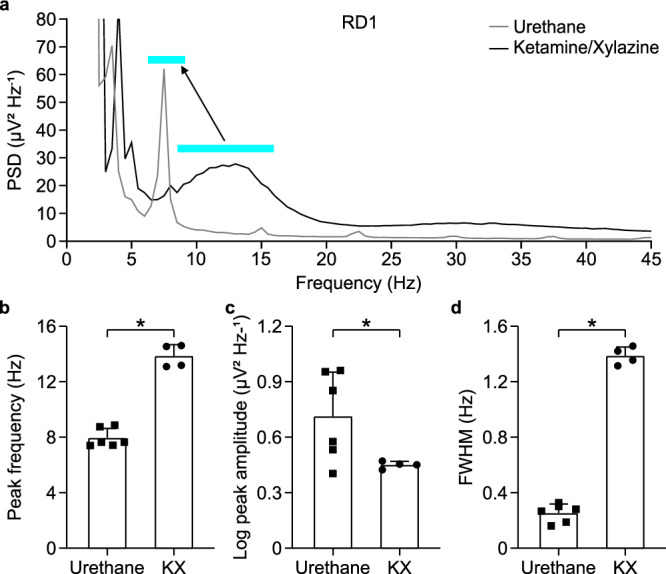
Periodic bursting activity in RD1 mice under mechanical ventilation and paralysis. **a** Eye average PSD from a RD1 mouse eye under urethane with mechanical ventilation and paralysis (gray) and under ketamine/xylazine mix with mechanical ventilation and paralysis (black). **b–d** Peak frequency (**b**), log peak amplitude (**c**) and FWHM (**d**) of the fitted Gaussian peaks corresponding to the periodic bursting activity (mean ± S.D.). Sample size is *n* = 6 eyes from *N* = 3 mice (81, 81, and 94 days old) for urethane and *n* = 4 eyes from *N* = 2 mice (78 days old) for ketamine/xylazine mix. Two-sided Mann-Whitney test for peak frequency: *p* = 0.0095, *u* = 0 and *r* = 0.81 (**b**). Two-sided Welch’s t test for log peak amplitude: *p* = 0.0428, t(5.10) = 2.68, 95% CI [−0.51, −0.01] and Cohen’s d = 1.55 (**c**). Two-sided Welch’s t test for FWHM: *p* < 0.0001, t(6.71) = 27.18, 95% CI [3.88, 4.63] and Cohen’s d = 17.50 (**d**). Source data are provided as a Source Data file.

After investigating RD10 mice, we next examined mice carrying the P23H rhodopsin mutation, one of the most common causes of autosomal dominant RP in humans. To date, however, bursting oscillatory activity has not been reported in this model even in explanted retinas. We, therefore, first tested for the presence of oscillatory activity ex-vivo. Retinal explants from five P23H mice (140–146 days old) recorded on microelectrode arrays exhibited a robust bursting oscillatory activity (Supplementary Fig. 7a). Similar to RD10 mice, the ex-vivo average PSD displayed a primary peak at ~5 Hz together with a second harmonic at twice the fundamental frequency, consistent with the non-sinusoidal nature of neuronal oscillations (Supplementary Fig. 7b, c). Based on these findings, we subsequently measured rsERG from four P23H mice (176, 177, 205, and 280 days old) under mechanical ventilation at 60 bpm. The in-vivo PSD exhibited an initial sharp peak at ~1 Hz corresponding to mechanical ventilation (green rectangle in Fig. 5e) and, consistently with the ex-vivo recordings, a second peak at ~5 Hz corresponding to retinal oscillatory activity (cyan rectangle in Fig. 5e). Following fitting, the oscillatory peak exhibited a mean peak frequency of 5.95 ± 1.16 Hz (±S.D.; Fig. 5f), mean log peak amplitude of 0.98 ± 0.41 µV^2^ Hz^-1^ (±S.D.; Fig. 5g), and mean FWHM of 1.20 ± 0.18 Hz (±S.D.; Fig. 5h). All parameters are significantly higher than zero (results of two-sided one sample t-tests in Fig. 5f–h). The mean goodness of fit is R^2^ = 0.98 ± 0.01 (±S.D.). In the P23H model, we also evaluated the effect of diazepam on oscillatory activity in-vivo (Fig. 5f–h). Similar to RD1 mice, diazepam administration (15 mg kg^-1^) significantly reduced the mean peak frequency from 5.95 ± 1.16 to 4.46 ± 0.53 Hz (±S.D.; Fig. 5f), the mean log peak amplitude from 0.98 ± 0.41 to 0.37 ± 0.32 µV^2^ Hz^-1^ ( ±S.D.; Fig. 5g) and the FWHM from 1.20 ± 0.18 to 0.76 ± 0.13 Hz (±S.D.; Fig. 6h). Results from two-way repeated measures ANOVA and Šídák’s multiple comparisons tests are reported in Supplementary Data 1. The mean goodness of fit is R^2^ = 0.99 ± 0.01 after diazepam (±S.D.).

Taken together, these results demonstrate that rsERG can detect retinal oscillatory activity across multiple RP mouse models exhibiting different dominant oscillation frequencies. Notably, P23H mice develop oscillatory rhythms similar to those observed in RD10 mice, consistent with the relatively slow degeneration profile shared by these two models. In addition, this dataset demonstrates that diazepam reduces oscillatory activity across distinct genetic backgrounds and disease-causing mutations. These findings highlight the translational relevance and robustness of rsERG across genetically diverse RP models.

In RD10 and P23H mice, the eye average PSD (Fig. 6) exhibited noticeably sharper peaks than those observed in RD1 mice (Figs. 1d, 2a, 3a). Several factors could account for this difference. One possibility is the use of mechanical ventilation combined with paralysis, which alters the breathing-related artifact. Another possibility is the difference in anesthetic protocol, namely ketamine/xylazine versus urethane. A third hypothesis is that the effect reflects intrinsic differences between retinal degeneration models (RD1 vs P23H and RD10). To disentangle these variables, we performed two additional experiments. First, we repeated the recordings in three RD1 mice (81, 81 and 94 days old) under urethane anesthesia combined with mechanical ventilation and paralysis, thereby controlling for the genetic model. Under these conditions, RD1 mice also exhibited a sharper oscillatory peak at a lower central frequency in the eye average PSD (gray in Fig. 6a). This finding argues against the spectral differences being solely model-dependent. Next, we performed the technically challenging experiment of recording under mechanical ventilation and paralysis while maintaining anesthesia with ketamine/xylazine in two additional RD1 mice (78 days old), in order to isolate the effect of urethane anesthesia itself. Under these conditions, the eye average PSD displayed an oscillatory peak closely resembling that observed in RD1 mice under ketamine/xylazine without mechanical ventilation or paralysis (black in Fig. 6a). FOOOF analysis confirmed that urethane anesthesia produced a statistically significant reduction in oscillatory peak frequency, an increase in oscillatory synchrony as indicated by reduced FWHM and an increase of log peak amplitude (statistical test results in Fig. 6b–d). The mean goodness of fit is R^2^ = 0.95 ± 0.04 for urethane and R^2^ = 0.95 ± 0.02 for ketamine/xylazine (± S.D.).

### Time-frequency analysis tracks oscillatory bursting activity in rsERG recordings

**Fig. 7:**
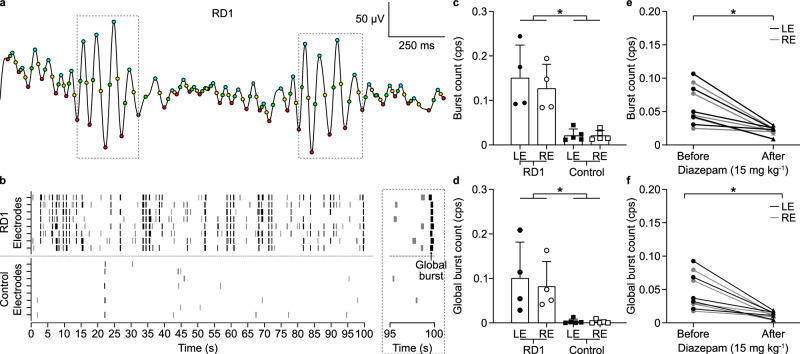
Time-frequency analysis on resting-state ERG tracks oscillatory activity in end-stage RD1 retinas in-vivo. **a** Example of a 3-s long 1–20 Hz filtered rsERG epoch from 1 microelectrode in one RD1 mouse eye. Neural oscillations are highlighted by cyan circles (peaks), red circles (troughs), and green/yellow circles (middle points). Dashed rectangles identify oscillations classified as bursts. **b** 100-s long representative raster plot of bursts for one RD1 and one control eye (representative excerpt from a 60-min long recording). Each row corresponds to one microelectrode of the array. The width of each rectangle corresponds to the burst duration. Black rectangles are global bursts. The inset shows an enlarged view highlighting one global burst. **c**, **d** Mean burst count (**c**) and mean global burst count (**d**) in RD1 and control mice (±S.D.). Sample size is *n* = 8 eyes from *N* = 4 mice (196-198 days old) for RD1 mice and n = 10 eyes from N = 5 mice (27 days old) for control mice. Full results of two-way ANOVA and Tukey’s multiple comparisons tests in Supplementary Data 1. RD1 vs Control: * p < 0.0001 for burst count (**c**) and * *p* = 0.001 for global burst count (**d**). **e**, **f** Mean burst count (**e**) and mean global burst count (**f**) before and after administration of diazepam at 15 mg kg^-1^. Sample size is *n* = 8 eyes from *N* = 4 mice (56–59 days old). Full results of two-way repeated measures ANOVA and Šídák’s multiple comparisons tests in Supplementary Data 1. Before vs After: * *p* = 0.0018 for burst count (**e**) and * *p* = 0.023 for global burst count (**f**). Source data are provided as a Source Data file.

In neuronal circuits, a distinct PSD peak is expected to reflect bursting neuronal oscillations in the temporal domain at the corresponding frequency. We therefore explored rsERG time series recorded from end-stage 28-weeks old RD1 mice and 4-weeks old C57BL/6 J control mice to identify oscillatory events using a recently described cycle-by-cycle analysis method^38^. Cycles were detected in band-pass filtered time series (1–20 Hz) by identification of troughs and peaks in the waveform (Fig. 7a). Cycles within the relevant frequency band (10–18 Hz) underwent further analysis. This range corresponds to the FWHM encompassing all oscillatory peaks identified in the previous PSD analysis (Fig. 1j). Presence of neuronal oscillatory activity was defined as segments containing at least three consecutive cycles exhibiting high values for amplitude consistency, period consistency, and monotonicity (see Methods^38^). These segments were classified as oscillatory bursts (dashed gray rectangles in Fig. 7a).

RD1 mice exhibit sustained bursting oscillations within the relevant frequency band across all microelectrodes of the flexible corneal neurotechnology (Fig. 7b). Averaging the burst count per second (cps) across the eight microelectrodes in each array yielded a mean burst count in RD1 mice of 0.14 ± 0.06 cps, significantly higher than control mice (±S.D.; results of two-way ANOVA and Tukey’s multiple comparisons tests in Supplementary Data 1). Moreover, bursts in RD1 mice are highly synchronous across electrodes, whereas the relatively rare bursts detected in control mice appear sporadic and spatially uncorrelated (Fig. 7b). To quantify this synchrony, we defined global bursts as bursts occurring simultaneously at least on four electrodes of the flexible corneal neurotechnology (black rectangles in Fig. 7b). RD1 mice exhibit a mean global burst count of 0.09 ± 0.06 cps, significantly higher than that observed in control mice (±S.D.; results of two-way ANOVA and Tukey’s multiple comparisons tests in Supplementary Data 1). Importantly, in RD1 mice the global burst count is not significantly different than the burst count (results of two-way ANOVA in Supplementary Data 1), whereas a significant difference is present in control mice regardless of the tested eye (results of two-way ANOVA and Tukey’s multiple comparisons tests in Supplementary Data 1). Together, these findings indicate that oscillatory bursts in RD1 mice arise from coherent retinal oscillations simultaneously detected across multiple microelectrodes rather than from isolated sporadic events as observed in control mice.

Finally, we evaluated burst count (Fig. 7e) and global burst count (Fig. 7f) before and after diazepam administration in RD1 mice (15 mg kg^-1^) using a frequency window from 7 to 15 Hz defined from the FWHM analysis in Fig. 3d. Diazepam treatment significantly reduced both burst count and global burst count in both eyes (results of two-way repeated measures ANOVA and Šídák’s multiple comparisons tests in Supplementary Data 1).

**Fig. 8:**
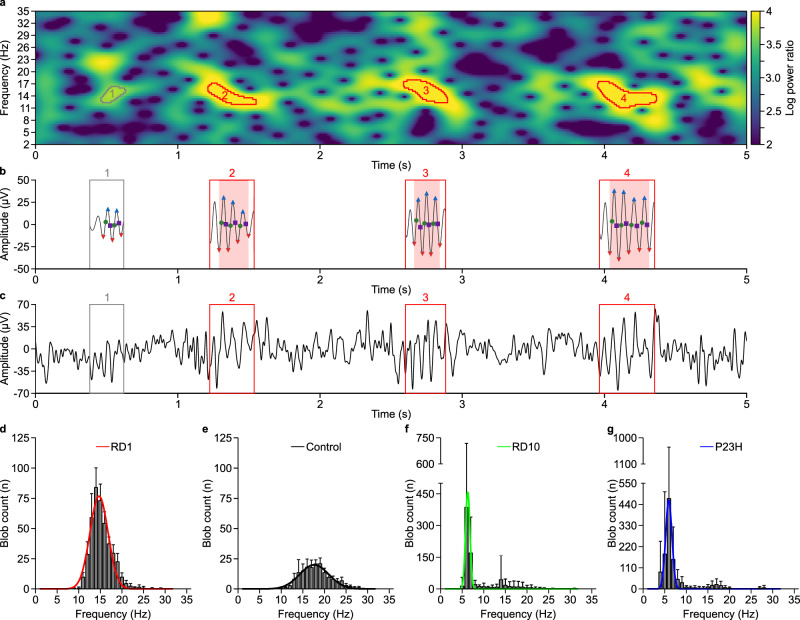
Spectral blob analysis in RP mouse models. **a** Representative 5-s long scalogram after subtraction of the aperiodic component from a rsERG recording in a RD1 mouse. A value from 2 to 4 indicates power from 100 to 10000 times the background (aperiodic) power at that frequency. The red and gray circles indicate the identified blob. **b** Cycle-by-cycle analysis applied to identified blobs determines blobs containing oscillatory bursts (in red) and non-oscillatory blobs (gray). **c** Mapping of blobs on the original unfiltered rsERG 5-s long epoch. **d–g** Mean blob count distribution for RD1 (**d**), control (**e**), RD10 (**f**), and P23H (**g**) mice (±S.D.). The solid lines are the gaussian interpolation of the distributions. Sample size is *n* = 8 eyes from *N* = 4 mice (196–198 days old) in RD1 mice, *n* = 10 eyes from *N* = 5 mice (101–102, 117–120, and 260 days old) in RD10 mice, *n* = 8 eyes from *N* = 4 mice (176, 177, 205, and 280 days old days old) in P23H mice, and *n* = 10 eyes from *N* = 5 mice (27 days old) in control mice. Source data are provided as a Source Data file.

Having established that time-frequency analysis of rsERG recordings can reliably identify bursting oscillatory activities within a known frequency range, we next explored a more holistic approach to detect neuronal oscillations across a broader frequency spectrum. Inspired by electroencephalography processing methods, we implemented spectral blob analysis to unambiguously detect the transient presence of neural oscillatory activity without a priori assumptions regarding central frequency or bursting characteristics. For this analysis, we used previously recorded dataset from control, RD1, RD10 and P23H mice. Given the strong correspondence between burst count and global count observed previously (Fig. 7c–f) recordings from the 8 microelectrodes of the flexible corneal neurotechnology were averaged to generate a single virtual channel, thereby simplifying the analysis procedure (Fig. 8).

Spectral blob analysis is performed on wavelet scalograms generated after subtraction of the aperiodic component (f^-1^), thereby emphasizing oscillatory signal components relative to the aperiodic background, which can otherwise dominate the low-frequency range. The resulting scalograms therefore represent log_10_ power ratios relative to the aperiodic baseline. Figure 8a shows a 5-s segment of an aperiodic-subtracted scalogram from an rsERG recording in an RD1 mouse eye, in which four blobs are identified. The gray blob (labeled 1) was excluded because it did not correspond to a bursting oscillatory activity, as it contains fewer than three consecutive valid cycles. In contrast, the three red blobs (labeled from 2 to 4) satisfied the oscillatory criteria and were retained (Fig. 8b). As in the previous cycle-by-cycle analysis, oscillatory behavior is defined as at least three consecutive cycles meeting criteria of amplitude consistency, period consistency, and monotonicity (see Methods). Finally, detected blobs were mapped back on the raw rsERG signal to verify that they were not driven by recording artifacts (Fig. 8c).

Blob count distributions from the four mouse groups (RD1, RD10, P23H and controls) are then compared (Fig. 8e-h). RD1 mice exhibit a dominant population of oscillatory blobs centered at ~15 Hz, consistent with previous PSD results (Fig. 1c). RD10 and P23H mice display a concentrated blob distribution within the 5–7 Hz, in agreement with earlier findings (Fig. 6). Lastly, control mice exhibit only a small population of oscillatory blobs within a relatively high frequency range (18–20 Hz). This observation is not entirely unexpected, as previous literature has reported spontaneous RGC discharge activity in explanted cat retinas at about ~25–35 Hz in darkness or under constant illumination^39^. Consistent with this observation, detailed spectral analysis of rsERG recordings from control mice frequently revealed a detectable peak within this frequency range (Supplementary Fig. 8). Similar high-frequency peaks are occasionally observed also in rsERG recordings from P23H and RD1 mice.

Next, blob count distribution in each mouse group was fitted with Gaussian functions (R^2^ = 0.91 for RD1 mice; R^2^ = 0.52 for RD10 mice; R^2^ = 0.34 for P23H mice; R^2^ = 0.80 for controls). Statistical analysis demonstrated that the four distributions could not be fitted by the same function (F-test: *p* < 0.0001, *F* = 53.22) indicating that the distributions differed significantly across groups. Specifically, when comparing RD10 and P23H mice, Gaussian amplitudes are not significantly different (F-test: *p* = 0.9879, *F* < 0.01), whereas both peak frequency and S.D. are significantly different (F-test: *p* = 0.0015 and *p* = 0.0062, *F* = 10.18 and *F* = 7.54; respectively for peak frequency and S.D.).

### Time-frequency blob analysis on resting-state ERG reveals neural oscillation in RP patients

Last, we assessed whether retinal oscillatory activity is present in patients with severe, end-stage RP. Patients exhibited no detectable light-evoked ERG responses, a best corrected visual acuity (BCVA) of less than 0.016 decimal (1.8 LogMAR) and a residual central visual field of less than 3 degrees. To this end, we enrolled 5 RP patients and 8 sighted participants (SPs) meeting the inclusion criteria (Supplementary Table 1).

Importantly, performing rsERG in humans presents several challenges. First, the flexible corneal neurotechnology used in mice is not approved for clinical use. ERG electrodes allowed in clinics are either contact lenses or threads, both of which provide only a single recording channel. Thread electrodes are substantially more comfortable and allow prolonged recordings, whereas contact lens electrodes provide more stable signals but are generally tolerated for only a few minutes because they require eyelid retractors. We chose ERG-Thread electrodes to enable 40-min rsERG recordings with closed eyelids. The disadvantage is the presence of strong motion artifacts due to relative micromovement between the eye and the thread. Second, unlike mice, participants are not anesthetized, further increasing the prevalence of eye motion-related artifacts in the rsERG signal. Third, human participants lack the predefined electrophysiological phenotypes available in genetically characterized mouse models, making analysis more challenging. Mouse models exhibit bursting oscillatory activity at ~5 Hz^11,12^, ~7 Hz^13^ or ~10 Hz^15^, in Royal college of surgeons (RCS) rats at ~2 Hz^40^, and at ~20 Hz in the retinas of Macaca fascicularis monkeys^14^. Whether comparable oscillatory activity exists in RP patients, and at which frequencies, remains unknown and may vary substantially across individuals.

**Fig. 9:**
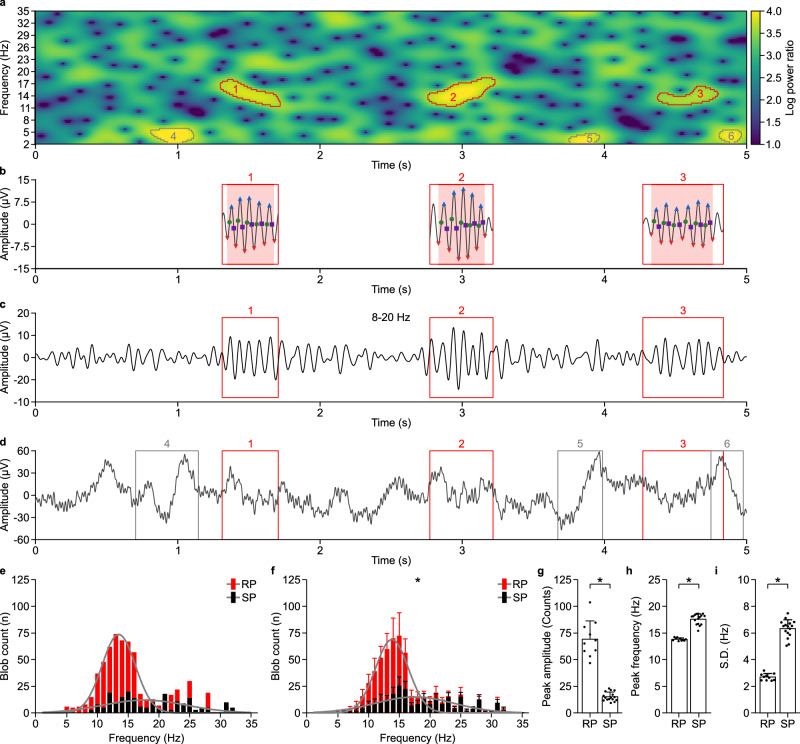
Spectral blob analysis in RP patients and SPs. **a** Representative 5-s long scalogram after subtraction of the aperiodic component from a rsERG recording in a RP patient. A value from 1 to 4 indicates power from 10 to 10000 times the background (aperiodic) power at that frequency. The red and gray circles indicate the identified blob. **b** Cycle-by-cycle analysis applied to identified blobs determines blobs containing oscillatory bursts (in red). **c** Mapping of blobs containing oscillatory bursts on the original filtered rsERG 5-s long epoch (8–20 Hz). **d** Mapping of blobs on the original unfiltered rsERG 5-s long epoch. **e** Representative blob count distribution for one eye from a RP patient (red) and one eye from a SP (black). The solid lines are the gaussian interpolation of the distributions. **f** Mean (±S.D.) blob count distribution for RP patients (red) and SPs (black). The solid lines are the gaussian interpolation of the distributions. **g–i** Quantification of mean (±S.D.) Gaussian parameters: peak amplitude (**g**), peak frequency (**h**) and S.D. (**i**). Sample size is *n* = 10 eyes from *N* = 5 RP patients and *n* = 16 eyes from *N* = 8 SPs. Two-sided Welch’s t test for peak amplitude: * *p* < 0.0001, t(9.71) = 9.86, 95% CI [−66.25, −41.74] and Cohen’s d = 4.94 (**g**). Two-sided Welch’s t test for peak frequency: * *p* < 0.0001, t(18.45) = 14.55, 95% CI [3.26, 4.35] and Cohen’s d = 4.76 (**h**). Two-sided Welch’s t test for S.D.: * *p* < 0.0001, t(20.98) = 19.56, 95% CI [3.24, 4.02] and Cohen’s d = 6.59 (**i**). Source data are provided as a Source Data file.

Given the strong agreement observed between spectral blob analysis and the PSD analysis in mice, we applied the spectral blob analysis pipeline to rsERG recordings from RP patients and SPs to determine whether neuronal oscillatory activity could be detected within specific frequency ranges (Fig. 9). Our mouse-validated approach, in which recordings from the 8 microelectrodes of the flexible corneal neurotechnology averaged together to generate a single virtual channel, translates naturally to the single-channel recordings obtained using ERG-Thread electrodes. Eyes have been considered an independent statistical unit for both inclusion criteria and data analysis. This strategy is consistent with our mouse results and the notion that in RP degeneration can be asymmetrical between the two eyes^41–43^. Raw rsERG recordings from human participants are noisier than those obtained from anesthetized mice (Fig. 9d). Nevertheless, correspondence between oscillatory blobs (Fig. 9a) and the associated temporal segments (Fig. 9b) could still be appreciated after band-pass filtering the rsERG signal around the frequency range of the detected blobs (e.g., 8–20 Hz in Fig. 9c). RP patients exhibit a significantly higher oscillatory blob count compared to SPs, and the resulting distributions are fitted by significantly different Gaussian functions (F-test: *p* < 0.0001, F = 441.3). Interestingly, the blob count distribution observed in RP patients most closely resembles the RD1 mouse phenotype, whereas SPs displayed a distribution similar to control mice. Gaussian parameters differ significantly between RP patients and SPs, including peak amplitude (Fig. 9g), peak frequency (Fig. 9h) and S.D. (Fig. 9i). Results of two-sided Welch’s *t* tests in Fig. 9g–i.

Overall, these findings demonstrate that retinal oscillatory activity is present in RP patients and is likely associated with retinal degeneration when compared with SPs. Despite the substantially lower signal quality relative to anesthetized mouse recordings, the rsERG-based methodology remained effective in patients, while also revealing substantial opportunities for further methodological refinement.

## Discussion

In this article we build on a long-standing thread in vision science initiated by Dräger and Hubel’s observation of a rhythmic 10–15 Hz activity of retinal origin in the superior colliculus and visual cortex in RD1 mice^9^. A similar activity was later seen in the superior colliculus of RCS rats, where Sauvé and colleagues reported a slower rhythm (~3 Hz)^44^, suggesting that abnormal signals are a common feature of degenerating retinas. Many ex-vivo studies using retinal explants confirmed these observations^10–14,25^ and importantly, linked this periodic bursting activity to changes in circuit wiring and altered network balance arising from retinal remodeling after photoreceptor loss^15–18^. Yet, so far, bursting oscillatory activity has never been measured in-vivo at the retinal level, neither in animal models nor in RP patients.

Clinically, light-evoked ERG and microperimetry are routinely used to assess residual retinal light sensitivity (rod/cone function), but no clinical method currently exists to evaluate functional circuit rewiring. Recordings ex-vivo from human retinas can, in principle, address this need^45,46^, but only in post-mortem tissue and therefore not in a clinically scalable or longitudinally accessible manner. Furthermore, RP donor tissues and non-human primate models^47,48^ remain scarce. This methodological gap motivated us to adapt a method commonly used in neurology. Resting-state electroencephalography measures intrinsic brain activity in the absence of sensory stimulation, similarly rsERG measures intrinsic retinal activity from the eye surface without external light stimulation. We propose that rsERG provides a non-invasive in-vivo approach capable of preserving both spectral and temporal characteristics of remodeled retinal activity, enabling repeated assessment across patients and throughout disease progression.

To maximize translational continuity between previous ex-vivo findings and the present in-vivo recordings, we reasoned that the most convincing validation strategy would be to test the proposed method directly against the field key ex-vivo studies. Neural oscillations are detected across all RP models we tested (RD1, RD10 and P23H) and their dominant frequency aligns with ex-vivo reports. We focused primarily on the RD1 and RD10 models because of their well-established time-course for degeneration, remodeling, and oscillatory activity as well as its pharmacologic modulation. However, we also examined the P23H model whose mutation is common in human RP, and whose time-course for anatomical degeneration and remodeling characteristics are known, but not its oscillatory activity. Using this work as predefined benchmarks, we developed an experimental strategy that also prioritized clinically meaningful applications, namely: longitudinal monitoring of disease progression, tracking responses to therapeutic interventions, and eventually, profiling discrete models of retinopathy to inform patient stratification and trial design.

Corneal rsERG is embedded in a signal-rich environment in which numerous non-retinal physiological sources can masquerade as retinal activity. Cardiorespiratory rhythms (respiratory rate at ~1–3 Hz and heart rate at ~3–7 Hz) introduce slow drifts and pulsatile artifacts; eye-movement and eyelid dynamics can produce large transients and broadband bursts; extraocular muscles contribute to high-frequency power with leakage into the lower frequency band; and interface factors (tear-film fluctuations, micro-slip, electrode polarization) add low-frequency wander and intermittent steps. The solution to many of these challenges is to stabilize the mouse with anesthesia. However, selecting the appropriate anesthesia is not straightforward given the risks of attenuating pure retinal signals. Published data using RD1 or RD10 retinal explants showed that periodic bursting activity could be reduced or abolished by drugs (e.g. AMPA/kainate receptor blockers, glycine, GABA, and benzodiazepines)^15,25^. For example, isoflurane acts through allosteric GABA_A_ receptor modulation and has been shown to alter light-evoked ERG waves^49^. Moreover, pentobarbital also potentiates GABA_A_ activity and significantly reduces light-evoked ERG signals^50^. On the contrary, the administration of NMDA receptor blocker alone did not result in any change in either spiking rate or local-field potential properties of the bursting oscillatory activity^15,25^. We therefore opted for a single dose of ketamine/xylazine, because ketamine acts primarily on NMDA receptors with limited effect on GABA_A_ receptors^51^ and xylazine is an agonist at alpha-2 adrenergic receptors. This anesthetic mixture should not interfere at synaptic level with the periodic rhythmic activity. Moreover, it has been shown that ketamine/xylazine inhibits volitional eye movements and oculomotor reflexes^52^, as well as spontaneous blinking^53,54^, and saccades^55^, but has little influence on light-evoked ERG^52^.

We further reasoned that, similar to EEG recordings under ketamine/xylazine anesthesia, rsERG recordings would likely still be contaminated with breathing and heart rate artifacts. Therefore, by using the RD1 mouse, whose retinal oscillations ex-vivo occur sufficiently outside the frequency band of mouse breathing and heart rates, we would increase our chances of capturing potential retinal signals. Nonetheless, we used a miniature stereotaxic head-restraint to further minimize residual head movements. To capture retinal oscillatory activity occurring within the ranges of breathing and heart rate frequency bands (in RD10 and P23H mice), we reasoned that additional immobilization of mouse movement would likely be necessary, and a strategy focused on further mitigating breathing artifacts would be required. We therefore used paralysis in conjunction with intubation and oxygen delivery under urethane anesthesia, to manually lower the breathing rate to 60 bpm (1 Hz) without causing acute hypoxia. This strategy was successful in unmasking oscillatory activity in both RD10 and P23H mice.

Somewhat unsurprisingly, urethane shifted the oscillatory network toward a slower frequency range (~7–8 Hz) and higher amplitude, consistent with earlier observations in the visual cortex of RD1 mice^9^. In the degenerating retina, oscillations arise from a Cx36-coupled ON cone bipolar–AII amacrine network in which Na⁺-dependent bursting and gap-junction coupling generate recurrent depolarization cycles^18,19^. Their frequency depends on how rapidly each cycle is reset by the balance between depolarizing currents (HCN and Na⁺) and outward K⁺ conductances^17,19^. Inhibitory input from GABAergic and glycinergic amacrine cells provides this reset; when it is blocked with gabazine or strychnine, bursts become longer, slower, and larger in amplitude, as shown in both RD1 and RD10 explanted retinas^15,35^.

Urethane is not a direct inhibitory receptor blocker, but it can reduce inhibitory efficacy by increasing K⁺ leak conductance and weakly modulating GABA_A_, NMDA, and AMPA receptors^56–58^. This lowers input resistance, reduces synaptic gain, and suppresses GABAergic transmission at the circuit level^59^, impairing the temporal precision of the inhibitory reset without abolishing it. Two-pore-domain potassium (K2P) channels are key regulators of resting membrane conductance and neuronal excitability, shaping the gain and temporal integration of synaptic inputs. Consistent with this, K2P channels are widely expressed in retinal neurons, including ganglion cells and multiple amacrine cells, providing a plausible substrate for circuit modulation^60,61^. Thus, urethane likely does not alter the core oscillator itself but rather shifts the network into a lower-gain state in which reset is less precise, slowing oscillatory activity as a consequence of altered network dynamics.

A key element to consider in the data comparison between mouse models is a difference among mouse strains, experimental ages and anesthesia methods. Although such differences might influence ocular physiology, they were necessary due to pathological differences and degeneration progression between models. RD1 is a fast degeneration model, while RD10 and P23H degenerate more slowly. Also the dominant frequency required, mechanical ventilation in the latter two models. However, these differences also highlight the strong cross-model convergence of the results, further supported by their alignment with ground-truth ex-vivo findings. Therefore, cross-model convergence highlights the robustness and generalizability of rsERG beyond single strain, specific age or anesthesia method.

One limitation of our study was the systemic delivery of diazepam, which is not acceptable in patients because of its widespread systemic effects. In addition, systemic delivery imposes relatively high doses to achieve an effect in retinal circuit modulation. Direct targeting of retinal GABA_A_ receptors would require intravitreal injections of diazepam (or an equivalent drug such as inhibitory amino acids) for ophthalmic use or alternatively the development of topical ophthalmic formulations capable of reaching the retina. We considered this strategy in mice, however, we were dissuaded by the potential confounds of using a non-ophthalmic systemic injectable diazepam pre-dissolved in 40% propylene glycol and 10% ethanol, both of which are known irritants to the corneal epithelium and the eye^62^. Moreover, topical ocular bioavailability is typically <5% because of tear turnover and corneal barriers^63^.

In this work we also succeeded in translating our preclinical findings into clinical data. Compared with the preclinical study, the clinical component was impacted by other challenges. Mice data were recorded having pre-established electrophysiological phenotypes, using a refined experimental protocol featuring sedation, head restraint, and in some cases paralysis and ventilation, and exploiting a flexible microelectrode array. None of these refinements could be applied to human participants. Large body and head motion were less impactful, since volunteers were comfortably seated on a reclining armchair and breathing rate in patients is much slower than mice. However, eye movements are far more prevalent compared to mice. A major limitation was the impossibility to use the flexible corneal neurotechnology (research device not approved for human use). The clinical electrode of choice (ERG-Threads) posed many challenges in long and continuous resting-state recordings. Because the reference electrode is conventionally fixed to the temple, the movement of the eye dipole generates baseline oscillations. Furthermore, the relative movements between the eye and the ERG-Thread likely introduces other movement artifacts. Finally, ERG-Threads provide single-channel recordings, thus excluding the use of conventional strategies for discerning artifacts (e.g. regression or independent component analysis).

For all these reasons, initial attempts to apply spectral analysis and FOOOF proved to be inefficient in identifying oscillatory peaks in end-stage RP patients. In contrast, we succeeded in designing a stringent analysis pipeline to circumvent limitations imposed by the use of approved electrodes. Time-frequency blob analysis resulted in a robust parameter to identify pathological neuronal oscillations. The five RP patients presented heightened resting-state oscillatory activity (blob counts) in a frequency range that most closely matches the mice RD1 phenotype, while SPs reported a count distribution matching control mice. Nevertheless, larger multiple cohorts of patients, grouped based on specific mutations or at least genes of interest, will likely be helpful to confirm robustness and clinical utility of the results.

In conclusion, this work establishes rsERG as a non-invasive way to quantify disease-linked retinal rhythms in-vivo and to follow their evolution over time in clinical context. rsERG offers a complementary biomarker to light-evoked ERG by reporting on remodeling-related dynamics rather than photoreceptor function alone. This methodology has immediate translational value and might have important clinically relevant implications by allowing a better understanding of retinal degeneration in patients and providing a crucial tool in clinical trials focused on sight restoration efforts. First, rsERG can stratify patients by oscillation burden/frequency and serve as a pharmacodynamic readout for agents that modulate inhibitory/excitatory balance (e.g., GABAergic or gap-junction modulators), enabling go/no-go decisions and dose finding. Second, rsERG can be used longitudinally to monitor disease progression and therapy response. Third, rsERG can function as a readiness marker for vision-restoration approaches such as optogenetics, prostheses, or regenerative medicine. Presence of neuronal oscillations might be an exclusion criterion in clinical trials since it reduces retinal excitability. On the other hand, strategy to block oscillations may expand the dynamic range for stimulation or residual signaling, as suggested by the diazepam-linked EC50 gains in cortical EEPs. Finally, because our pipeline is agnostic to specific signal changes and relies on convergent temporal-spectral features, it is adaptable to other retinal diseases in which remodeling is suspected to generate circuit alterations (e.g., AMD, diabetic retinopathy, etc.).

More broadly, the eye is a window to the brain because the retina and optic nerve develop from the diencephalon during embryonic growth, making them integral to the central nervous system^64^. Hence, resting activity in the retina may encode latent physiological state, like cortical resting-state dynamics, primarily in neurology and psychiatry but also in any systemic physiology that perturbs retinal circuits. Also, the retinal resting activity might contain a wealth of information about health and disease, which is unexplored in both clinical practice and daily life.

## Methods

### Ethical authorizations for animal experiments

Animal experiments ex-vivo were performed according to Austrian laws governing animal experimentation. The research animal facility was approved by the responsible Federal Ministry of Education, Science and Research: BMWFW-66.009/0403-WF/V/3b/2014. Animal experiments in-vivo were conducted according to the animal authorization VD3930 approved by the Direction générale de l’agriculture, de la viticulture et des affaires vétérinaires of the Canton de Vaud in Switzerland.

### Mouse lines and husbandry

All the experiments were carried out during the day cycle. Mice were kept in a 12 h day/night cycle (7am–7pm) with access to food and water ad libitum. Temperature and humidity were controlled, respectively 23 °C and 40–60%. For the entire duration of the experiment, the health condition was evaluated three times a week. Experiments were performed in female C57BL/6 J (control) mice (*N* = 13 mice, 27–70 years old), homozygous female C3H/HeNRj (RD1) mice (*N* = 36 mice, 29–198 years old), homozygous male/female B6.CXB1-Pde6b^rd10^/J (RD10) mice (*N* = 5 mice, 101–260 years old), and homozygous male/female B6.129S6(Cg)-Rho^tm1.1Kpal^/J (P23H) mice (*N* = 4 mice, 140–146 years old). Control and RD1 mice were purchased from Janvier Labs (France). RD10 and P23H mice were bred in our facility.

### Recording of resting-state ERG in mice

Mice were anesthetized with a mix of ketamine (87.5 mg kg^–1^) and xylazine (10 mg kg^–1^) injected intraperitoneally. Depth of anesthesia was verified every 15 min by absence of pedal and palpebral reflexes. Supplementary 10% doses of ketamine were administered as required. Eyes were protected with artificial tears (Lacryvisc, Alcon) during preparation and recordings. Body temperature was maintained at 37 °C with a feedback‑controlled heating pad (Right‑Temp, Kent Scientific). Animals were secured in a miniature stereotaxic frame (SGM‑3, Narishige) mounted on a vibration‑isolation platform (Thorlabs) inside a light‑tight aluminum enclosure (Thorlabs). A flexible microelectrode array (8 microelectrodes plus a large ground electrode in each array)^31^ was positioned onto each eye and covered with artificial tears. Tape was used to reposition the orientation of the whiskers, which prevented potential movement artifacts caused by contact with the recording array. Each array was then connected to an intan head-stage (C3324, Intan Technologies) and connected to an Open Ephys acquisition board. Independent input chains were used to minimize inter-ocular cross talk. Signals were acquired, band-pass filtered (0.1–500 Hz) and digitized at 2000 Hz via the Open Ephys GUI (v0.6.5). Baseline stability and absence of large movement artifacts was confirmed for 5 min before starting data acquisition. Mice were not dark-adapted but kept in dark within an aluminum enclosure (XE25C11D, Thorlabs) during recordings.

### Recording of resting-state ERG under mechanical ventilation in mice

Mice were anesthetized with an intraperitoneal injection of urethane (1.6 g Kg^-1^) delivered in two half-doses 10 min apart. 100% O_2_ was provided throughout the procedure. Peripheral oxygen saturation, heart rate and breathing rate were monitored continuously using a MouseOx Plus neck collar (Starr Life Sciences). Cervical fur was shaved and then removed with depilatory cream. With 100% O_2_ delivery and a heating pad beneath the body to keep body temperature at 37 °C, the animal was placed supine on a mouse intubation platform (Kent Scientific). The mouse was secured to the intubation platform by a surgical thread hooked through the upper incisors^65^. The forepaws were gently taped flush against the platform. This maneuver flattened the thorax, lowering the sternum and thereby widening the viewing corridor to the glottis. Mucus was cleared with a cotton swab (Micron, Purity Swabs). The light from a surgical microscope was directed at the ventral surface of the mouse near the neck to provide trans‑illumination, and a lens magnifier enabled visualization of the epiglottis and arytenoid cartilage “flaps”^66^. The tongue was retracted ventrally with a dental spatula to better visualize the glottis. A saline lubricated 20 G endotracheal catheter (ETT-2010, Kent Scientific) was rested on the lower incisors and advanced towards the epiglottis; immediately before entry its tip was deflected ventrally to avoid the esophagus. Correct tracheal placement was confirmed by smooth spontaneous breathing. The catheter hub was wedged beneath the lower incisors to lock the placement. After 5 min of spontaneous breathing, the mouse was transferred prone onto the heating pad and the catheter hub was connected to a calibrated mechanical ventilator (RoVent, Kent Scientific) supplemented with 100% O_2_. Parameters were set as: 120 bpm, 10 cmH_2_O peak pressure, and 0.5 ml tidal volume. Effective ventilation was verified by synchronous thoracic movements and stable SpO_2_ > 98%. Paralysis was induced with an intraperitoneal injection of pancuronium bromide (0.4 mg kg^-1^) to abolish spontaneous respiration^67^. Five minutes after injection, the flexible microelectrode arrays were positioned on the cornea. The breathing rate was then lowered by 10 bpm every 30 s until 60 bpm, then recording was started as described above. Mice were not dark-adapted but kept in dark within an aluminum enclosure (XE25C11D, Thorlabs) during recordings.

### Pharmacology

Diazepam was used at its stock concentration (injectable solution, 5 mg ml^-1^). Injections were delivered intraperitoneally with 29 G insulin syringes at volumes scaled to body mass: 1 µl g^-1^ for the 5 mg kg^-1^ cohort and 3 µl g^-1^ for the 15 mg kg^-1^ cohort. Oxybuprocaine 0.4% was applied topically.

### Recording of ex-vivo explanted retinas

Eyes were enucleated after animal euthanasia by cervical dislocation. The cornea and lens were removed, and the eye was hemisected to extract the retina. The vitreous was then carefully removed from the retina surface. A retinal segment of approximately 2.5–3.5 mm^2^ was placed RGCs side down onto a 60-electrode glass chip (60MEA200/30iR-ITO, Multi Channel Systems). Prior to use, each chip was cleaned with a 5% Tickopur R60 detergent solution, plasma treated (ZEPTO, Diener electronic) and coated with 200 µl of poly-L-lysine (1 mg ml^-1^, MW 150–300 kDa, Sigma-Aldrich) to promote tissue adhesion. Throughout preparation and recording, samples were maintained in carbogenated (95% O2, 5% CO2) Ames’ medium (Sigma-Aldrich) supplemented with NaHCO3 (1.9 mg ml^-1^). After placement, retinas were dark-adapted for 45 min before recording, then retinal activity was recorded continuously for 1 h in dark. Data were amplified (MEA60-System headstage, Multi Channel Systems), low-pass filtered (12.5 kHz) and digitalized at 25 kHz (Multi Channel Experimenter, v2.21.1). To compute the PSD, signals were band-pass filtered (1–45 Hz) using a Butterworth filter, then downsampled from 25 kHz to 100 Hz via polyphase resampling. PSD was estimated using Welch’s method with 5-s windows and 50% overlap. For each retina, the average PSD was calculated as the mean across electrodes showing good retinal contact: presence of detectable spiking activity from the 300–3500 Hz band-pass filtered traces.

### Corneal stimulation and electrically evoked potentials

Mice were anesthetized with a mix of ketamine (87.5 mg kg^–1^) and xylazine (10 mg kg^–1^) injected intraperitoneally. Depth of anesthesia was verified every 15 min by absence of pedal and palpebral reflexes. Supplementary 10% doses of ketamine were administered as required. Eyes were protected with artificial tears (Lacryvisc, Alcon) during preparation. The scalp was infiltrated with 2% lidocaine and excised after 5 min. The periosteum was cleared with 3% H_2_O_2_. Three holes were drilled and three stainless‑steel screws (0.8 mm in diameter) were inserted: one reference electrode (–2.0 mm AP relative to bregma, +1.0 mm ML), and two recording electrodes over the primary visual cortices (+1.0 mm AP relative to lambda, ±1.5 mm ML). Leads were wrapped around the screws and connected to two independent differential amplifiers (Model 3000, A–M Systems). Signals were amplified and band-pass filtered (1–300 Hz) and digitized at 1000 Hz (CED Micro1401, Signal v6). Corneal stimulation was generated by an isolated pulse stimulator (Model 2100, A–M Systems) and delivered using ERG‑JET electrodes (LCK Technologies) filled with artificial tears. The return electrode was a subcutaneous needle electrode inserted below the eye. Monophasic cathodal pulses of 100 µs duration were presented at 0.5 Hz repetition rate with stimulus amplitudes pseudo‑randomized in an alternating low–high–low sequence (in µA): 50, 1500, 100, 1250, 200, 1000, 300, 950, 350, 900, 400, 850, 450, 800, 500, 750, 550, 700, 600, and 650. Only in RD1 mice, three more current amplitudes were included (in µA): 2000, 1500, and 2500. Twenty repetitions were averaged per intensity. After the baseline series, diazepam (5 or 15 mg kg^-1^) was intraperitoneally injected, and the sequence was repeated after a 10-min break. N1-P1 complexes were identified in a timewindow from 10 to 90 ms after the stimulus onset from averaged responses for each current amplitude. Peak-to-peak amplitude was quantified as the difference from N1 to P1. Mice were not dark-adapted but kept in dark within an aluminum enclosure (XE25C11D, Thorlabs) during recordings.

### Ethical authorizations for human experiments

Experiments with RP patients and SPs were conducted according to the Declaration of Helsinki and the ethical standards of the Swiss Federal Human Research Act (HRA). Experiments received approval from the Cantonal Commission for Ethics in Research (CER-VD). Approval ID 2023-01518 for RP patients and 2024-00999 for SPs. Informed consent was obtained from all human research participants.

### Study participants

Eight SPs (1 male and 7 females; Mean ± S.D. Age 30.75 ± 6.86, range 24–46 years) and five RP patients (3 males and 2 females; Mean ± S.D. Age 55.80 ± 12.40, range 40-71 years) were enrolled in the study after confirming the eligibility criteria. For RP patients, inclusion/exclusion criteria were set as follows. Inclusion: 18 years old or more; no response to full-field ERG; BCVA less than 0.016 decimal (1.8 LogMAR); visual field less than 3 degrees. Exclusion: history of glaucoma, retinal detachment, eye trauma, central retinal artery or vein occlusion; patients who have undergone vitrectomy surgery; inability to follow the procedures, e.g. due to language problems, psychological disorders, dementia, etc; patients not tolerating being in the dark for 60 min. For SPs, inclusion/exclusion criteria were set as follows. Inclusion: 18 years old or more; BCVA higher than 0.8 decimal (0.1 LogMAR); visual field more than 130 degrees. Exclusion: history of glaucoma, retinal detachment, eye trauma, central retinal artery or vein occlusion; candidates who have undergone vitrectomy surgery within the last 6 months; presence of silicon oil (applied to the eye); epileptic patients; inability to follow the procedures, e.g. due to language problems, psychological disorders, dementia, etc; candidates not tolerating being in the dark for 60 minutes.

### Recording of resting-state ERG in human subjects

After signing the informed consent form and confirming inclusion criteria, SPs and RP patients were conducted in the rsERG examination room. Subjects were seated on a reclining armchair and ERG-thread electrodes were positioned on both eyes. For each eye, a reference electrode was placed on each temple and a common ground electrode on the forehead. Dark-adaptation was performed for 30 min and then the registration session started for 40 min. Data were acquired with a E3 system (Diagnosys LLC), 1–300 Hz band-pass filtered, and sampled at 1000 Hz.

### Spectral analysis of resting-state ERG

Recordings were converted into 5-s long epochs, imported into MNE-Python^68^, and band-pass filtered (1–45 Hz) using a linear-phase FIR filter. PSD was estimated using Welch’s method with 5-s windows (epochs) and 50% overlap. Then, artifact rejection was performed on PSD using two criteria. PSDs exceeding by 3 times the channel’s mean PSD in the 1–3 Hz frequency range were excluded due to high breathing-related motion artifacts. Then, to remove broad-band noisy PSD, seven 2–Hz wide bands (from 7 to 8 Hz to 19–20 Hz) were assessed against the channel’s mean PSD. PSDs exceeding by 10 times the channel’s mean PSD in at least four bands were excluded. Finally, the PSDs were first averaged within each microelectrode, and then the eight PSDs from each microelectrode were averaged together into an average PSD for the entire array. Average PSDs were passed to the FOOOF algorithm to separate the aperiodic background from discrete oscillatory peaks^33^. The aperiodic component was fitted in knee mode, which adds a bending parameter to the parameters (exponent and offset) of a simple straight line (linear f^-1^) in log-log space.

### Burst detection

Band-pass filtered (1–20 Hz) 5-s long epochs were processed via cycle-by-cycle analysis algorithm detecting cycles using according to three criteria: (i) the amplitude of the cycle computed as the average voltage difference between the peak and the two adjacent troughs, (ii) the period of the cycle defined as the time between the two troughs, and (iii) the rise-decay symmetry defined as the fraction of the period that was composed of the rise time^38^. For further analysis, only cycles within specific frequency bands were considered. Bursts were defined as a sequence of at least three consecutive retained cycles having high-quality metrics for monotonicity, amplitude consistency, and period consistency (e.g. ≥ 0.6). Amplitude consistency of a cycle is the relative difference in the rise and decay voltage. Period consistency of a cycle is the maximal relative difference between the cycle’s period and the period of the adjacent cycles. Monotonicity is the fraction of instantaneous voltage changes that are positive during the rise phase and negative during the decay phase^38^. For multi-electrode mice data, global bursts were identified as bursts occurring synchronously at least on 4 electrodes of the flexible corneal neurotechnology.

### Time-frequency blob analysis

Continuous Morlet wavelet transforms were computed on 5-s long epochs using MNE-Python^68^. Wavelet cycles were scaled with frequency to maintain constant temporal resolution across the frequency axis. A wavelet guard criterion was applied to exclude frequencies whose wavelet duration exceeded the 5-second epoch window, giving a practical lower frequency limit of 2 Hz. To visualize oscillatory activity independently of the broadband f^-1^ spectral decay inherent to neural signals, an epoch-wise aperiodic correction was applied. The power-law component of each epoch’s power spectral density was estimated by fitting a straight line in log-log space^33,69^ and subtracted from the log-transformed wavelet power at each time-frequency point. Spatially contiguous regions of elevated power in the corrected scalogram (blobs) were identified by thresholding and connected-component analysis of the wavelet time-frequency representation^70^. Briefly, pixels exceeding the 95th percentile of the scalogram power distribution were labeled using 8-connectivity, and candidate regions were accepted based on minimum area, duration, and estimated cycle count. Each connected component was characterized by two frequency span definitions. The mask span was the full frequency range of all suprathreshold pixels. The core span was the frequency range of pixels whose detection value exceeded 65% of the component peak detection value. The core span was used as the primary descriptor for all gating and downstream analyses because it excludes low-power peripheral pixels that extend the mask span without contributing to the main oscillatory structure. Blobs with > 15 Hz frequency span (> 10 Hz core span) were excluded as broadband artifacts. To separate merged detections, a watershed algorithm split components with multiple power peaks, and narrow bridges between sub-regions were severed computationally. Each blob was characterized by its time boundaries, frequency span, peak frequency, and estimated cycle count. Detected blobs could be refined interactively by the analyst. For each detected blob, the raw signal was band-pass filtered to the blob’s frequency span using a zero-phase Butterworth filter applied bidirectionally to avoid phase distortion. The filtered signal was segmented into putative cycles using trough-to-trough boundaries following the cycle-by-cycle analysis framework^38^, such that each segment corresponded approximately to one period of the oscillation of interest. For each cycle, primary features were computed including period, instantaneous frequency (inverse period), amplitude, and rise-decay symmetry, as described in the previous section. These primary features were then used to derive additional metrics that quantify the regularity and waveform structure of each cycle: amplitude consistency, period consistency and monotonicity. Blobs were retained only if they contained valid cycles, satisfying inclusion criteria. Specifically, cycles were required to fall within the blob-defined frequency band, to exceed a minimum amplitude threshold relative to the local noise floor, and to meet thresholds for amplitude consistency, period consistency, and monotonicity. For amplitude consistency and period consistency, thresholds were defined as a function of the blob’s center frequency, imposing stricter requirements at higher frequencies where oscillations are expected to exhibit greater cycle-to-cycle regularity. These criteria enforce the expectation that oscillatory activity exhibits local regularity in both amplitude and timing, as well as smooth waveform structure with consistent monotonic rise and decay phases. Contiguous runs of valid cycles were identified, and sequences containing at least three consecutive valid cycles were considered candidate bursts. To ensure that detected bursts reflected dominant oscillatory structure within the blob, a segment was classified as a burst only if at least 70% of cycles within the blob interval were part of the detected burst. Also, candidate burst should not contain signal artifacts, defined by three automated criteria based on robust z-scores: (i) large-amplitude deflections exceeding |z | ≥ 5.0 for more than 10 ms; (ii) sharp transient spikes detected via the signal first-difference exceeding |z | ≥5 .0 for more than 2 ms; and (iii) anomalous slow drift in the 0.5–1.0 Hz band. Accepted bursts were characterized by their duration, cycle count, mean instantaneous frequency, and mean amplitude. This approach integrates conventional spectral and cycle-based analyses: rather than applying a fixed frequency band across the entire recording, each blob’s detected frequency span directly defined the bandpass filter used in subsequent burst analysis. This ensures that cycle-by-cycle characterization operates within a region where oscillatory power has already been independently confirmed in the time-frequency domain.

### Data and Statistical analysis

Data preprocessing was performed in Python (version 3.11.7) using MNE-Python (version ≥ 1.6). Statistical analyses and graphical representation in Prism (GraphPad, version 10.2.3). Parametric assumptions were tested with the D’Agostino & Pearson normality test. Statistical significance was accepted at *p* < 0.05.

### Reporting summary

Further information on research design is available in the Nature Portfolio Reporting Summary linked to this article.

## Supplementary information

Supplementary Information

Description of Additional Supplementary Files

Supplementary Data 1

Reporting Summary

Transparent Peer Review file

## Source data

Source Data

## Data Availability

The data generated in this study are provided in the Source Data file. The data used in this study are available in the Zenodo database [10.5281/zenodo.17632802]. Source data are provided with this paper.

## Code availability

The analysis code used in the study is available in the Zenodo database [10.5281/zenodo.20030417].
