# Supplementary material for: Resting-state electroretinography reveals pathological retinal oscillations in retinitis pigmentosa mice and patients
Source: Nat Commun. 2026 Jul 10;17:8537. doi: 10.1038/s41467-026-75520-9 (PMC13482292; doi:10.1038/s41467-026-75520-9)
Supplement: Supplementary file 1 [file 41467_2026_75520_MOESM1_ESM.pdf]

SUPPLEMENTARY INFORMATION

Resting-state electroretinography reveals pathological retinal oscillations in retinitis pigmentosa mice and patients

David G. Litvin[1], Alexia Boizot[1], Andrea Corna[2], Aurélie Navarro[3], Josiane Keller[3], Marc O. Heuschkel[4], Adrien Roux[4], Günther Zeck[2], Hoai Viet Tran[5,6] and Diego Ghezzi[1,*]

[1] Ophthalmic and Neural Technologies Laboratory, Department of Ophthalmology, University of Lausanne, Hôpital ophtalmique Jules-Gonin, Fondation Asile des Aveugles, Lausanne, Switzerland

[2] Biomedical Electronics and Systems, Institute of Biomedical Electronics, Technische Universität Wien, Vienna, Austria

[3] Centre d'Investigation Clinique, Department of Ophthalmology, University of Lausanne, Hôpital ophtalmique Jules-Gonin, Fondation Asile des Aveugles, Lausanne, Switzerland

[4] Tissue Engineering Group, School of Engineering, Architecture and Landscape of Geneva (HEPIA), University of Applied Sciences and Arts Western Switzerland (HES-SO), Geneva, Switzerland

[5] Oculogenetics unit, Department of Ophthalmology, University of Lausanne, Hôpital ophtalmique Jules-Gonin, Fondation Asile des Aveugles, Lausanne, Switzerland

[6] Centre for Gene Therapy and Regenerative Medicine, King's College London, London, UK

* Corresponding author: info@ghezzilab.org

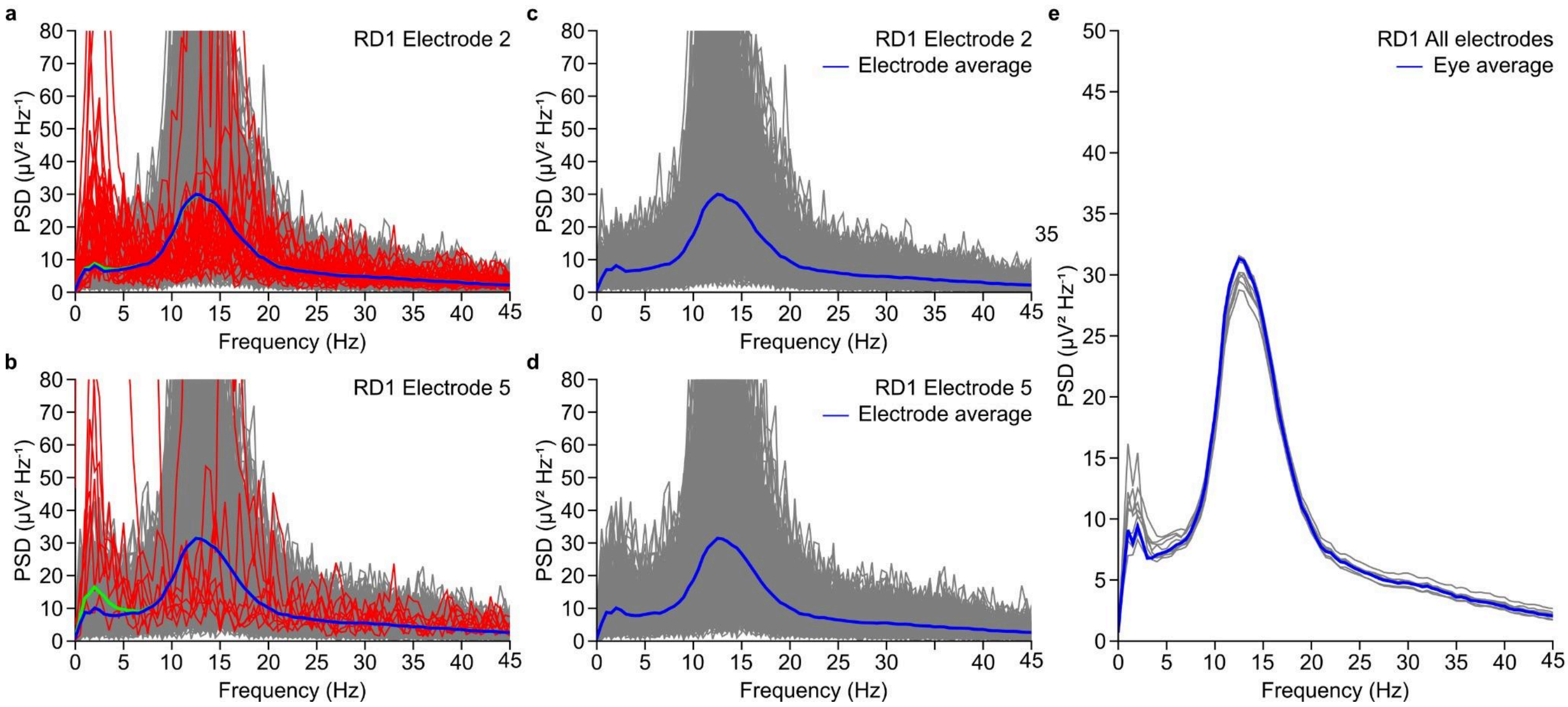

Supplementary Fig. 1 | Processing of resting-state ERG data in the spectral domain. a,b PSDs are computed across the entire recordings for each microelectrode of the flexible array and artifact rejection is performed. Grey epochs are conserved, red epochs are rejected. The green PSD is the electrode average before rejection and the blue is after rejection. Panel **a** shows an electrode in which the electrode average PSD is not affected by artifact rejection, while panel **b** shows an electrode in which artifact rejection successfully decreases the breathing artifact but does not alter the high-frequency peak. **c,d** Selected PSDs after artifact rejection in grey with the electrode average PSD in blue (from panels **a** and **b** respectively). **e** Overlay of the electrode average PSDs from the eight microelectrodes in grey with their eye average PSD in blue.

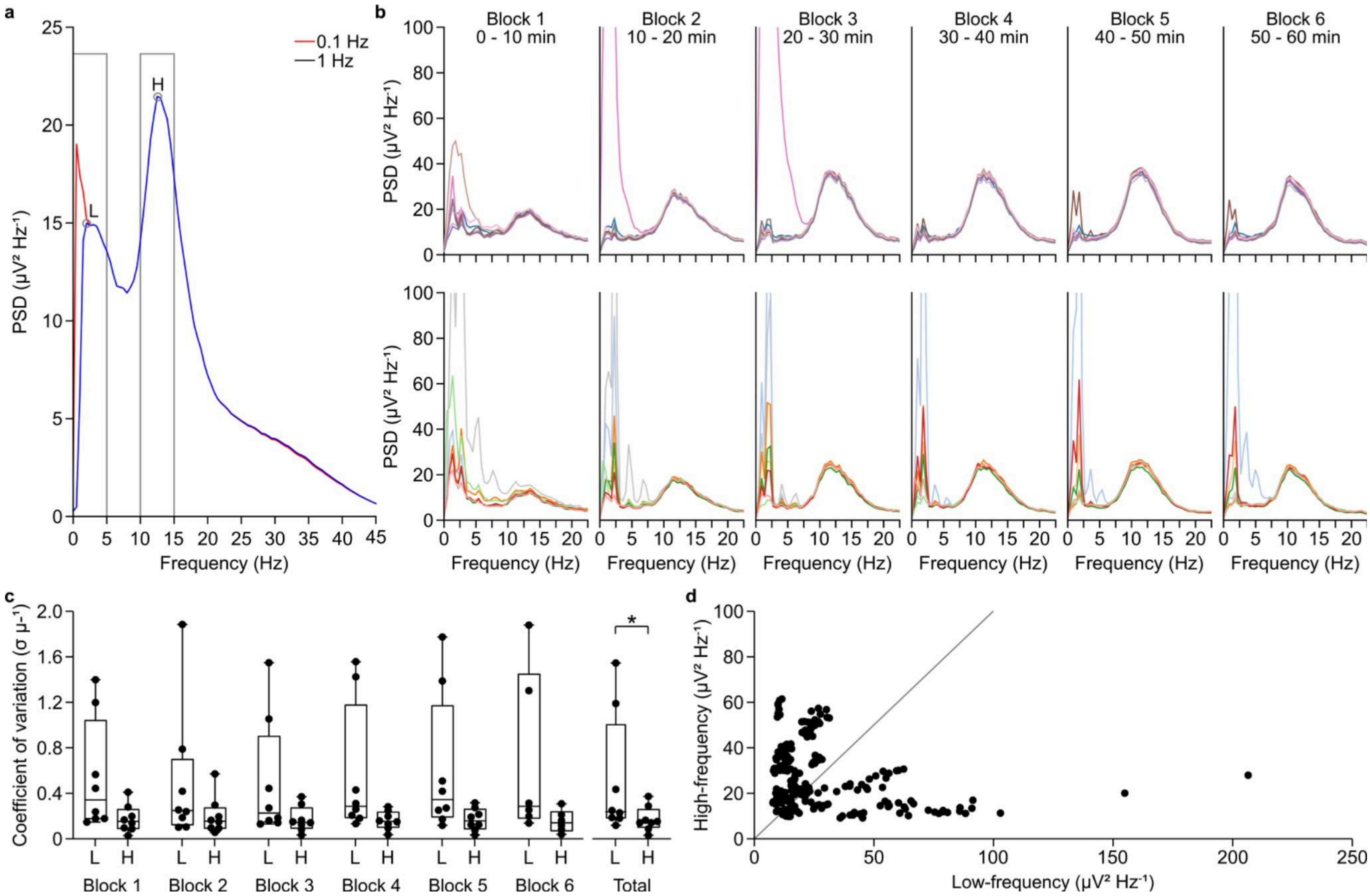

Supplementary Fig. 2 | Low-frequency and high-frequency PSD peak amplitudes are not correlated. a Eye average PSD from one RD1 mouse eye band-pass filtered at 1-45 Hz (blue) and 0.1-45 Hz (red). L and H represent respectively the PSD peak amplitude in the 0-5 Hz window (low-frequency peak) and 10-15 Hz window (high-frequency peak). **b** Electrode average PSDs from one RD1 mouse (left eye top and right eye bottom) divided into six consecutive blocks of 10 min each. **c** Coefficient of variation (S.D. / mean) for both low-frequency (L) and high-frequency (H) PSD peak amplitudes in each measured RD1 mouse eye. The coefficient of variation is calculated across electrode average PSD peak amplitudes within each block of 10 min and for the entire recording (Total). Low-frequency PSD peak amplitudes have much higher variability compared to high-frequency PSD peak amplitudes. Boxes extend from the 25th to 75th percentiles, whiskers extend from the min to max values, and the line is the median value (one-sided paired t-test for total analysis: * $p = 0.0425$, $t(7) = 2.01$, 95% CI [-0.75, 0.06] and Cohen's $d = 0.65$). Sample size is n = 8 eyes from N = 4 mice (196-198 days old). **d** Correlation plot between low-frequency and high-frequency PSD peak amplitudes across all electrodes recording from all mice recorded. Sample size is 8 microelectrodes from n = 8 eyes from N = 4 mice (196-198 days old). Spearman's correlation coefficient $r = -0.18$ ($p = 0.0061$). The grey line is the unity line. Source data are provided as a Source Data file.

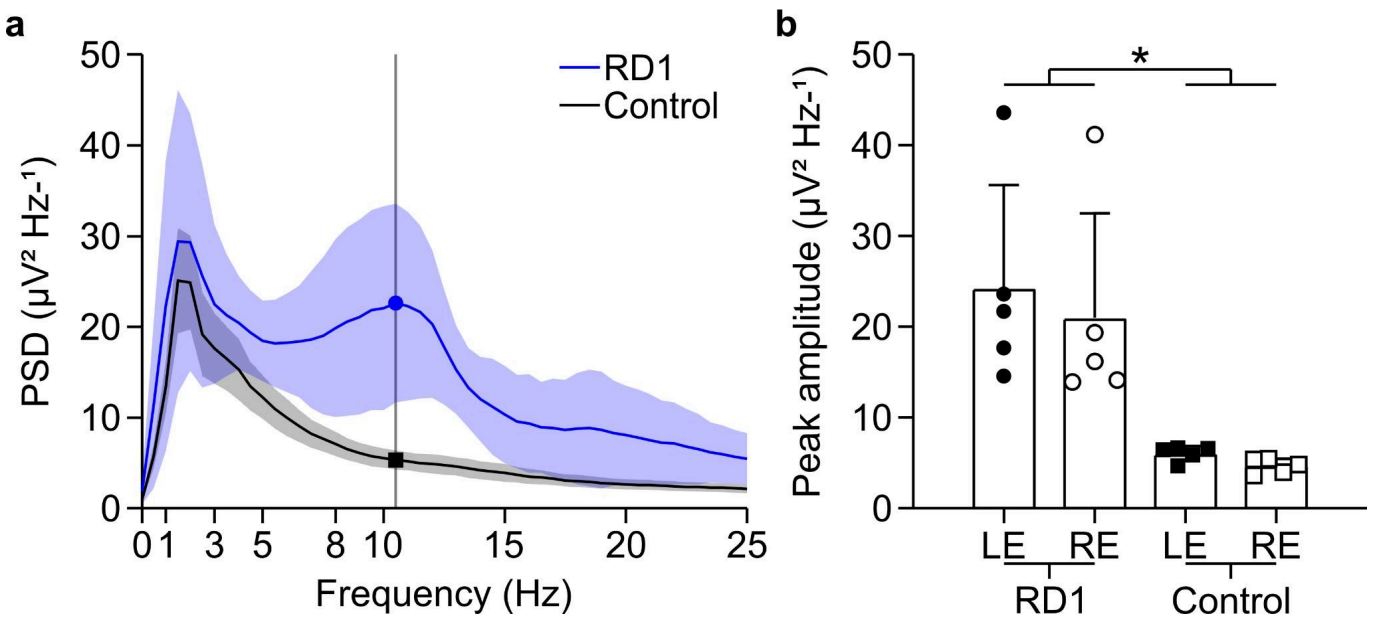

Supplementary Fig. 3 | Resting-state ERG in age-matched RD1 and control mice. a Mean eye average PSDs from RD1 mice in blue and control mice in black. The shaded area indicates ± S.D. Sample size is n = 10 eyes from N = 5 mice (29 days old) for RD1 mice and n = 10 eyes from N = 5 mice (27 days old) for control mice. **b** Mean PSD peak amplitude at 10.5 Hz in RD1 and control mice (± S.D.). Sample size is n = 10 eyes from N = 5 mice (29 days old) for RD1 mice and n = 10 eyes from N = 5 mice (27 days old) for control mice. Results of two-way ANOVA. Interaction: p = 0.7980, F(1, 16) = 0.07, Partial Eta Squared η_p^2 = 0.00; LE/RE: p = 0.5290, F(1, 16) = 0.41, Partial Eta Squared η_p^2 = 0.03; RD1/control: * p = 0.0002, F(1, 16) = 22.58, Partial Eta Squared η_p^2 = 0.59. Results of Tukey's multiple comparisons test: RD1:LE vs control:LE p = 0.0129; RD1:LE vs control:RE p = 0.0075; RD1:RE vs control:LE p = 0.0459; RD1:RE vs control:RE p = 0.0270; RD1:LE vs RD1:RE p = 0.9178; control:LE vs control:RE p = 0.9928. Source data are provided as a Source Data file.

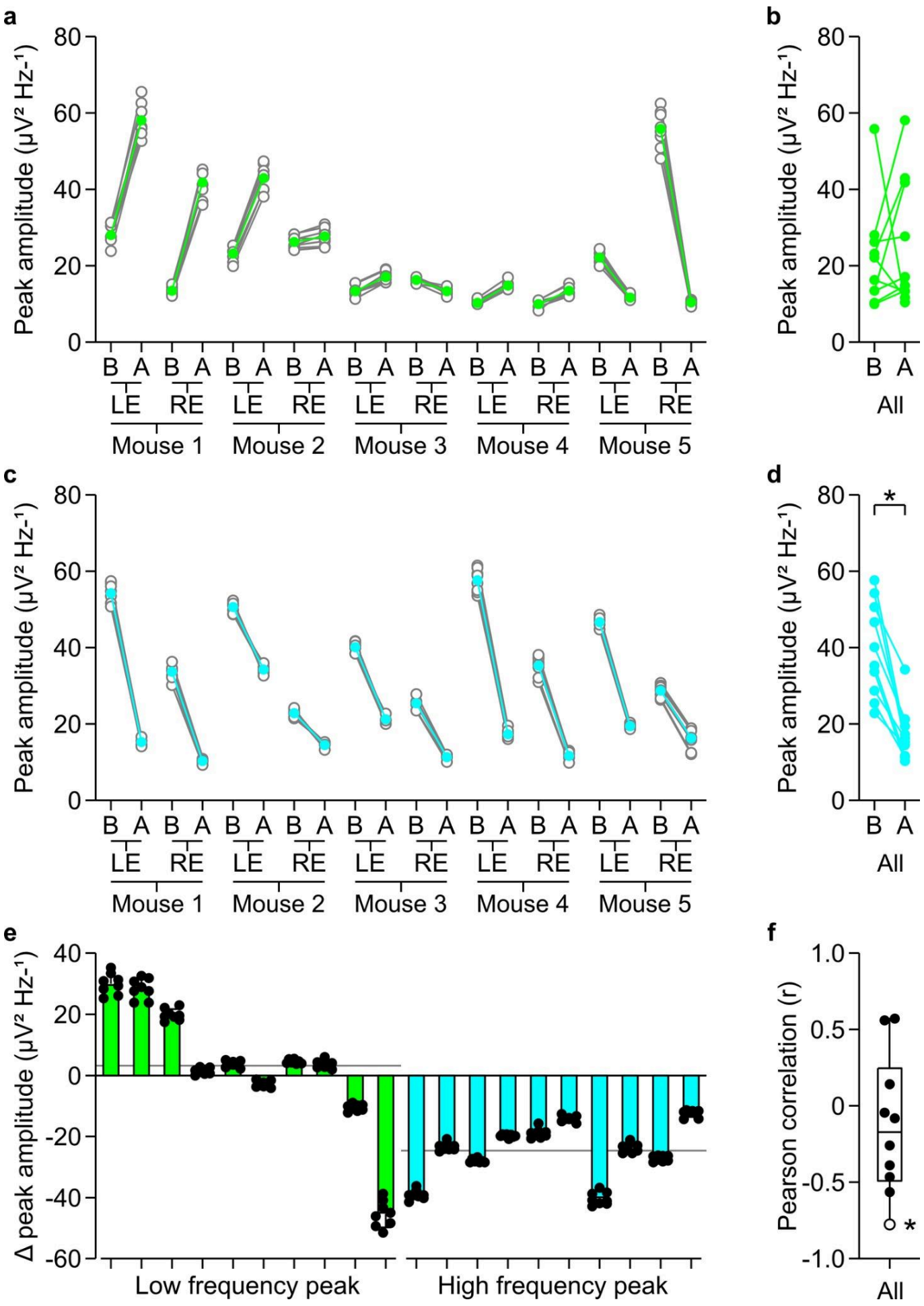

Supplementary Fig. 4 | Diazepam-induced variations in the low- and high-frequency PSD amplitudes are not correlated. a Change in the low-frequency PSD peak amplitude before (B) and after (A) diazepam administration at 15 mg kg^{-1} in RD1 mice. The open circles are the changes in the electrode average PSDs. Green circles are the average changes. **b** Pooled average changes from all eyes are not statistically significant (two-sided paired t-test: $p = 0.6456$, $t(9) = 0.48$, 95% CI [-12.24, 18.75] and Cohen's $d = 0.15$). **c** Change in the high-frequency PSD power peak amplitude before and after diazepam administration of diazepam at 15 mg kg^{-1}. The open circles are the changes in the electrode average PSDs. Cyan circles are the average changes. **d** Pooled average changes from all eyes are statistically significant (two-sided paired t-test: * $p < 0.0001$, $t(9) = 6.56$, 95% CI [-30.04, -14.70] and Cohen's $d = 2.08$). **e** Differential changes in PSD peak amplitude before and after administration of diazepam at 15 mg kg^{-1} for the low-frequency breathing-related artifact (green) and the high-frequency peak (cyan). Each circle corresponds to one electrode average PSD, while the bar is the mean ± S.D. The grey lines are the average differential changes across all eyes for each peak. **f** Pearson correlation coefficient between the low-frequency and high-frequency differential changes for each eye. A two-sided t-test on the Pearson coefficient determines a statistically significant correlation only for one eye out of 10 (open circle: $r = -0.77$, * $p = 0.0241$ and $t = -3.00$). All other correlations are not statistically significant (black circles: at least $p \geq 0.1379$). Boxes extend from the 25th to 75th percentiles, whiskers extend from

the min to max values and the line is the median value. Sample size is n = 10 eyes from N = 5 mice (56-59 days old) in all panels. Source data are provided as a Source Data file.

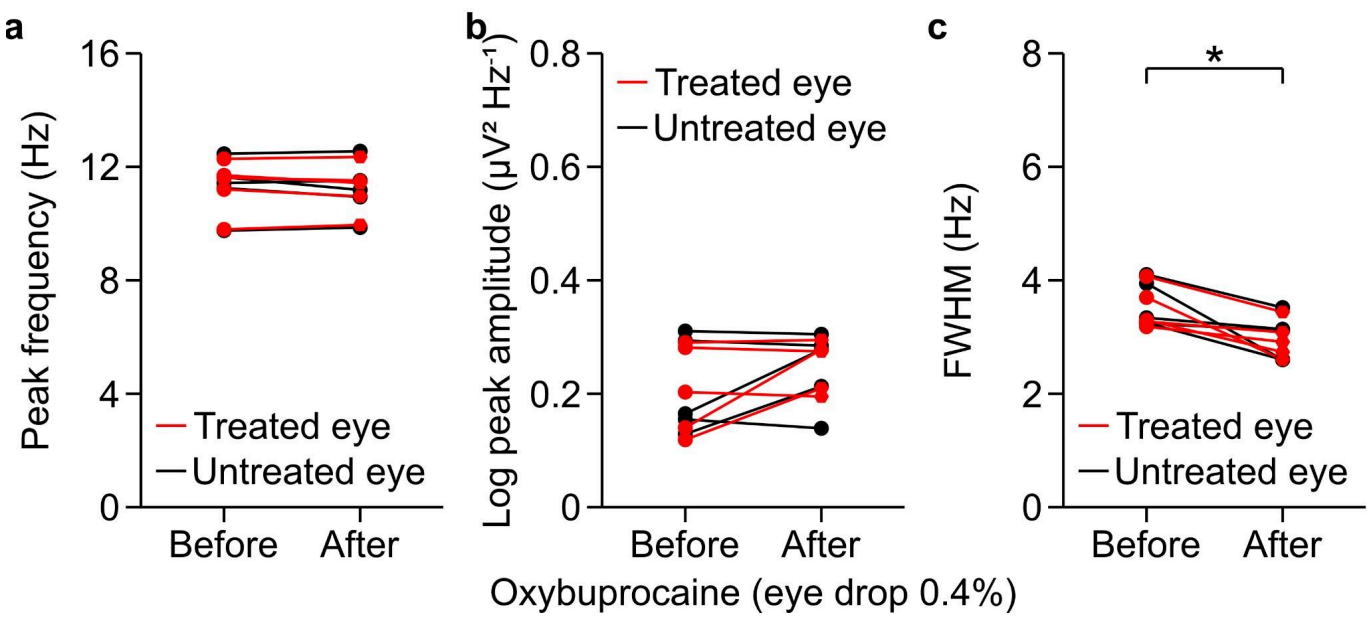

Supplementary Fig. 5 | Oxybuprocaine administration in-vivo. a Peak frequency of the fitted Gaussian peaks before and after oxybuprocaine administration in one eye (in red). Results of two-way repeated measures ANOVA. interaction: $p = 0.8814$, $F(1, 8) = 0.02$, Partial Eta Squared $\eta_p^2 = 0.00$; before/after: $p = 0.2679$, $F = 1.42$, Partial Eta Squared $\eta_p^2 = 0.15$; treated/untreated eyes: $p = 0.9687$, $F = 0.01$, Partial Eta Squared $\eta_p^2 = 0.01$. **b** Log peak amplitude of the fitted Gaussian peaks before and after oxybuprocaine administration in one eye (in red). Results of two-way repeated measures ANOVA. Interaction: $p = 0.1458$, $F(1, 8) = 0.06$, Partial Eta Squared $\eta_p^2 = 0.01$; before/after: $p = 0.0937$, $F = 3.62$, Partial Eta Squared $\eta_p^2 = 0.31$; treated/untreated eyes: $p = 0.9733$, $F = 0.00$, Partial Eta Squared $\eta_p^2 = 0.00$. **c** FWHM of the fitted Gaussian peaks before and after oxybuprocaine administration in one eye (in red). Results of two-way repeated measures ANOVA. Interaction: $p = 0.9173$, $F(1, 8) = 0.01$, Partial Eta Squared $\eta_p^2 = 0.00$; before/after: * $p = 0.0030$, $F = 1764$, Partial Eta Squared $\eta_p^2 = 0.69$; treated/untreated eyes: $p = 0.7806$, $F = 0.08$, Partial Eta Squared $\eta_p^2 = 0.02$. Results for the Šídák's multiple comparisons test: treated before/after $p = 0.0397$; untreated before/after $p = 0.0316$. Sample size is n = 5 eyes from N = 5 mice per group (49-51 days old) in all panels. Source data are provided as a Source Data file.

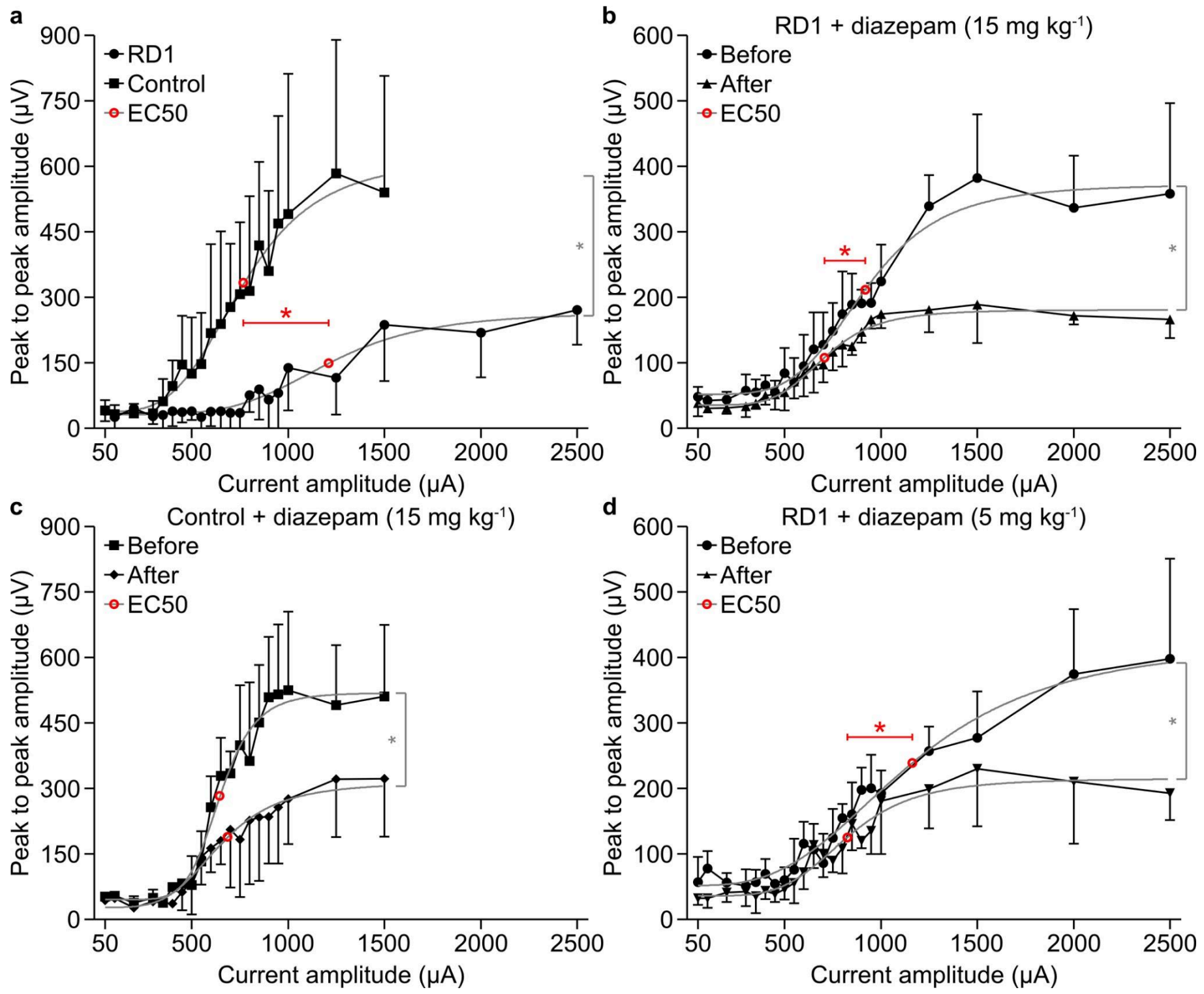

Supplementary Fig. 6 | Absolute input-output responses. a Mean input-output response curves for RD1 mice (± S.D.; n = 9 eyes from N = 5 mice, 62-72 days old) and control mice (± S.D.; n = 10 eyes from N = 5 mice, 57-70 days old). Regressions are significantly different (F-Test: * p < 0.0001, F(4, 390) = 79.93.84). EC50 values are also significantly different (767.7 µA for controls and 1211 µA for RD1 mice; F-Test: * p = 0.0043, F(1, 390) =7.85). **b** Mean input-output response curves for RD1 mice (± S.D.; n = 5 eyes from N = 5 mice, 55-57 days old) before and after diazepam administration at 15 mg kg^{-1}. Regressions are significantly different (F-Test: * p < 0.0001, F(4, 212) = 51.96). EC50 values are also significantly different (919.5 µA to 707.5 µA; F-Test: * p = 0.0001, F(1, 212) = 15.69). **c** Mean input-output response curves for control mice (± S.D.; n = 3 eyes from N = 3 mice, 55 days old) before and after diazepam administration at 15 mg kg^{-1}. Regressions are significantly different (F-Test: * p < 0.0001, F(4, 112) = 20.82). EC50 values are not significantly different (643.7 µA before and 686.3 µA after; F-Test: p = 0.6522, F(1, 112) = 0.20). **d** Mean input-output response curves for RD1 mice (± S.D.; n = 5 eyes from N = 5 mice, 55-59 days old) before and after diazepam administration at 5 mg kg^{-1}. Regressions are significantly different (F-Test: * p < 0.0001, F(4, 212) = 24.15). EC50 values are also significantly different (F-Test: 1162 µA before and 825.1 µA after; * p = 0.0006, F(1, 212) = 12.11). In panels **a-d**, the grey lines are the four parameter logistic regressions. Torus rings indicate the EC50 points. The red bars indicate a significant change in the EC50 value. Source data are provided as a Source Data file.

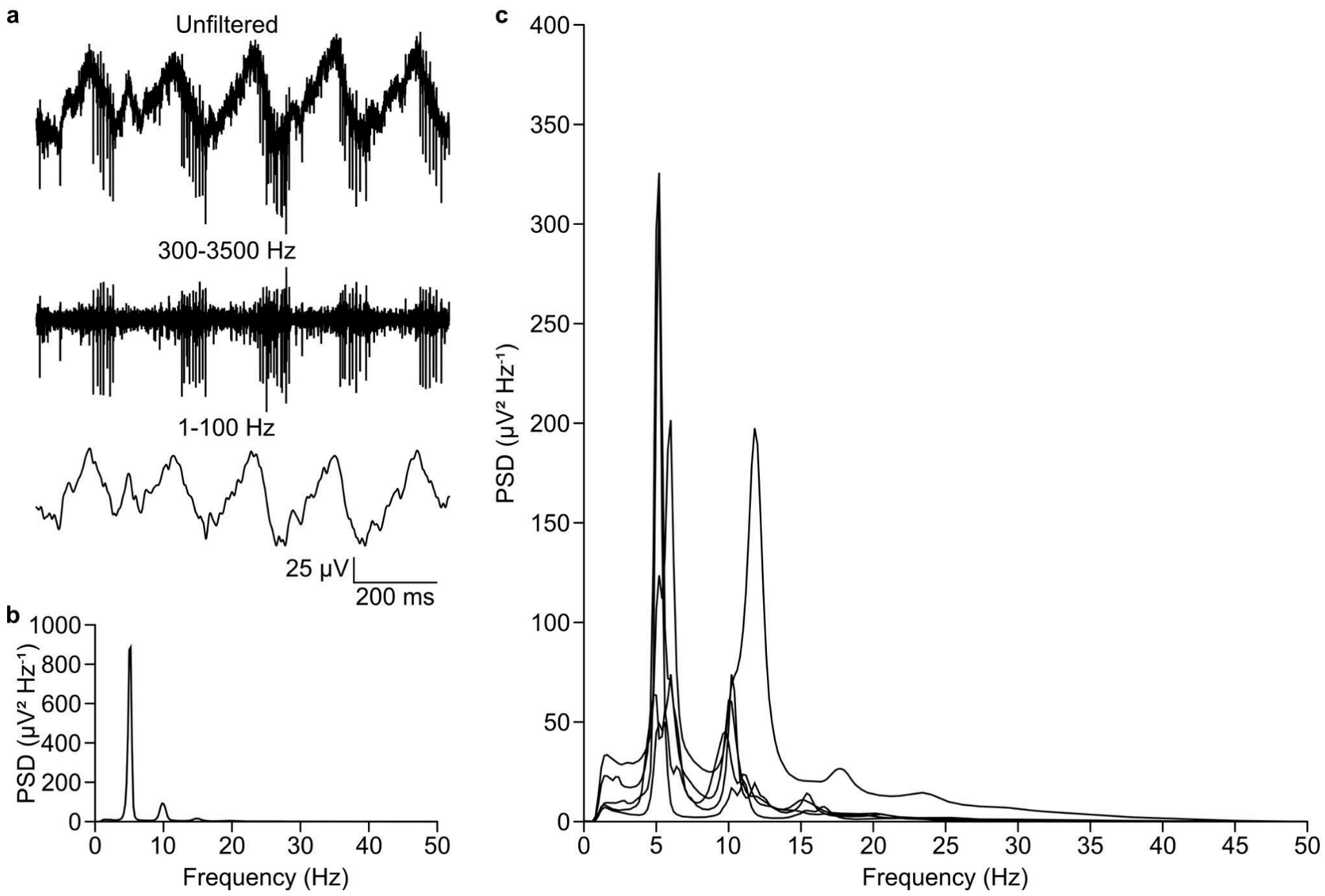

Supplementary Fig. 7 | Periodic bursting activity in P23H retinal explants. a 1-s long recording from a single microelectrode from a P23H retinal explant. The signal is presented unfiltered, 300-3500 Hz band-pass filtered to highlight neural spikes from RGCs and 1-100 Hz band-pass filtered to highlight neural oscillations. **b** PSD corresponding to the representative signal in **a**. **c** Overlay of average PSDs from P23H explanted retinas (n = 5 retinas from N = 5 animals). Each PSD is the average of all PSDs obtained from electrodes in the whole array.

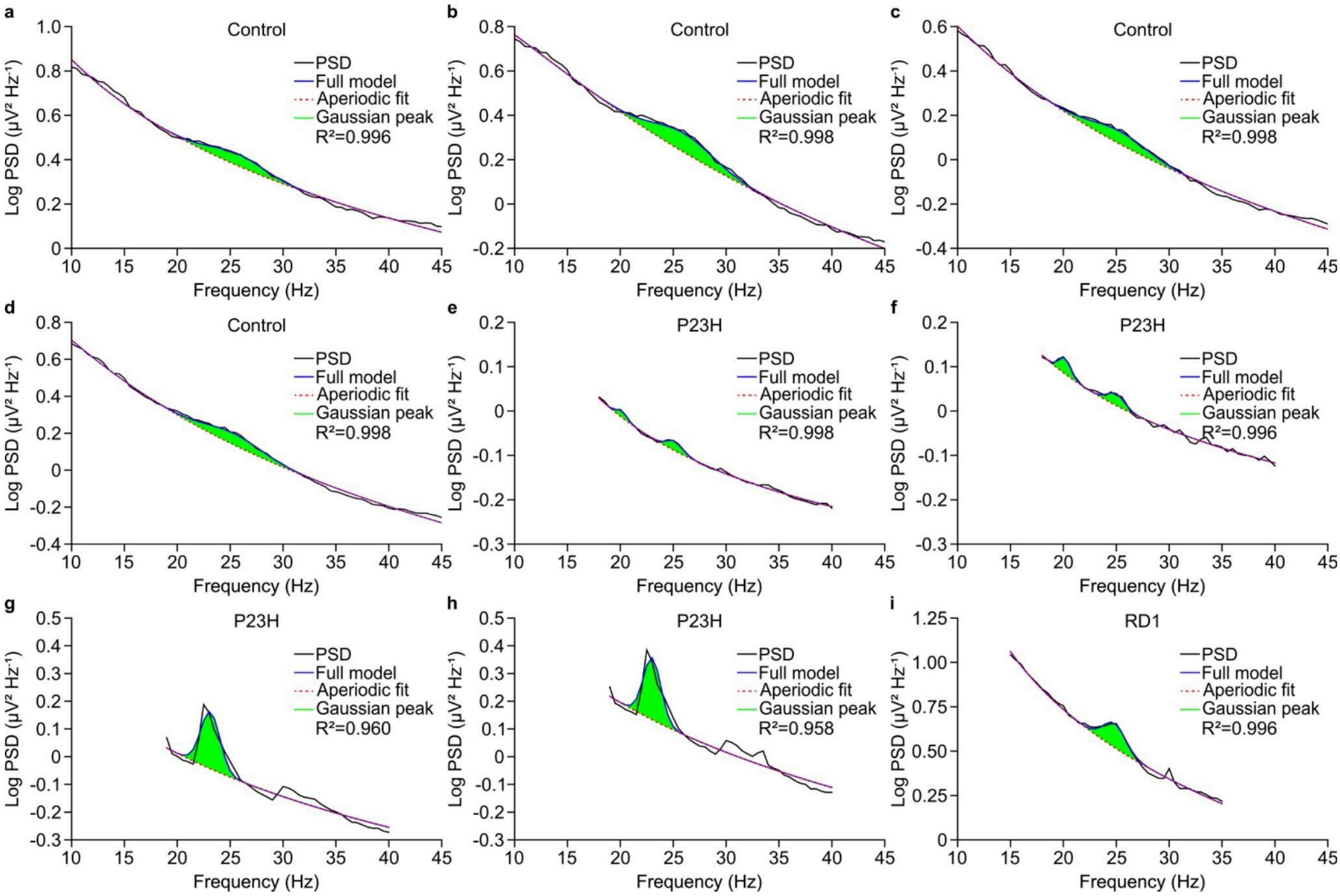

Supplementary Fig. 8 | Example of oscillatory peaks in the PSD at 20-25 Hz. Fitted eye average PSDs from control mouse eyes (**a-d**), P23H mouse eyes (**e-h**) and a RD1 mouse eye (**i**) showing a Gaussian peak at around 25 Hz of peak frequency.

Supplementary Tab. 1 | Summary of enrolled participants. SP indicates sighted participants and RP indicates retinitis pigmentosa patients. L indicates left eye and R indicates right eye. LP indicates BCVA at light perception level, HM at hand motion level and CF at counting fingers.

	**SP1**		**SP2**		**SP3**		**SP4**		**SP5**		**SP6**		**SP7**		**SP8**	
**Eye**	L	R	L	R	L	R	L	R	L	R	L	R	L	R	L	R
**BCVA (decimal)**	1.6	1.6	1.6	1.6	1.25	1.25	1.25	1.25	1.6	1.25	1.6	1.6	1.25	1.25	1.6	1.6
**IOP**	22	22	21	23	13	14	20	20	20	20	21	21	15	17	13	15
**VF > 130 °**	Y	Y	Y	Y	Y	Y	Y	Y	Y	Y	Y	Y	Y	Y	Y	Y

	**RP1**		**RP2**		**RP3**		**RP4**		**RP5**	
**Eye**	L	R	L	R	L	R	L	R	L	R
**BCVA (decimal)**	LP	LP	HM	CF	LP	CF	LP	HM	LP	HM
**VF < 3 °**	Y	Y	Y	Y	Y	Y	Y	Y	Y	Y
