# Supplementary material for: Resting-state electroretinography reveals pathological retinal oscillations in retinitis pigmentosa mice and patients
Source: Nat Commun. 2026 Jul 10;17:8537. doi: 10.1038/s41467-026-75520-9 (PMC13482292; doi:10.1038/s41467-026-75520-9)
Supplement: Supplementary file 2 [file 41467_2026_75520_MOESM2_ESM.pdf]

Description of Additional Supplementary Files

Supplementary Data 1: Results of two-way ANOVA analysis
