# Supplementary material for: Resting-state electroretinography reveals pathological retinal oscillations in retinitis pigmentosa mice and patients
Source: Nat Commun. 2026 Jul 10;17:8537. doi: 10.1038/s41467-026-75520-9 (PMC13482292; doi:10.1038/s41467-026-75520-9)
Supplement: Supplementary file 3 [file 41467_2026_75520_MOESM4_ESM.pdf]

nature portfolio

Corresponding author(s): Diego Ghezzi

Last updated by author(s): Jun 25, 2026

Reporting Summary

Nature Portfolio wishes to improve the reproducibility of the work that we publish. This form provides structure for consistency and transparency in reporting. For further information on Nature Portfolio policies, see our Editorial Policies and the Editorial Policy Checklist.

Statistics

For all statistical analyses, confirm that the following items are present in the figure legend, table legend, main text, or Methods section.

n/a	Confirmed	
☐	☒	The exact sample size (*n*) for each experimental group/condition, given as a discrete number and unit of measurement
☐	☒	A statement on whether measurements were taken from distinct samples or whether the same sample was measured repeatedly
☐	☒	The statistical test(s) used AND whether they are one- or two-sided<br>*Only common tests should be described solely by name; describe more complex techniques in the Methods section.*
☒	☐	A description of all covariates tested
☐	☒	A description of any assumptions or corrections, such as tests of normality and adjustment for multiple comparisons
☐	☒	A full description of the statistical parameters including central tendency (e.g. means) or other basic estimates (e.g. regression coefficient) AND variation (e.g. standard deviation) or associated estimates of uncertainty (e.g. confidence intervals)
☐	☒	For null hypothesis testing, the test statistic (e.g. *F*, *t*, *r*) with confidence intervals, effect sizes, degrees of freedom and *P* value noted<br>*Give P values as exact values whenever suitable.*
☒	☐	For Bayesian analysis, information on the choice of priors and Markov chain Monte Carlo settings
☒	☐	For hierarchical and complex designs, identification of the appropriate level for tests and full reporting of outcomes
☐	☒	Estimates of effect sizes (e.g. Cohen's *d*, Pearson's *r*), indicating how they were calculated

Our web collection on statistics for biologists contains articles on many of the points above.

Software and code

Policy information about availability of computer code

Data collection	Ex-vivo retina data were collected with the Multi Channel Experimenter (Multi Channel Systems, V 2.21.1).<br>In-vivo retina data were collected with the Open-Ephys GUI (V 0.6.5)<br>In-vivo electrically evoked potentials were collected with the Signal software (CED, V 6)<br>In-vivo rsERG data in patients were collected with the E3 system (Diagnosys)
Data analysis	Data preprocessing was performed in Python (version 3.11.7) using MNE-Python (version ≥ 1.6).<br>Statistical analyses and graphical representation in Prism (GraphPad, V 10.2.3).<br>Custom analysis code used in the study is available in the Zenodo database [https://doi.org/10.5281/zenodo.20030417].

For manuscripts utilizing custom algorithms or software that are central to the research but not yet described in published literature, software must be made available to editors and reviewers. We strongly encourage code deposition in a community repository (e.g. GitHub). See the Nature Portfolio guidelines for submitting code & software for further information.

Data

Policy information about availability of data

All manuscripts must include a data availability statement. This statement should provide the following information, where applicable:

- Accession codes, unique identifiers, or web links for publicly available datasets
- A description of any restrictions on data availability
- For clinical datasets or third party data, please ensure that the statement adheres to our policy

The data generated in this study are provided in the Source Data file. The data used in this study are available in the Zenodo database [https://doi.org/10.5281/zenodo.17632802].

Research involving human participants, their data, or biological material

Policy information about studies with human participants or human data. See also policy information about sex, gender (identity/presentation), and sexual orientation and race, ethnicity and racism.

Reporting on sex and gender	Sex is reported based on participant self-reports. Participants were not recruited/included according to their sex.<br>Sex, age, ethnicity, sociodemographic background and life style are not relevant factors for the study.
Reporting on race, ethnicity, or other socially relevant groupings	Grouping based on race, ethnicity, or other socially relevant groupings was not performed.<br>Sex, age, ethnicity, sociodemographic background and life style are not relevant factors.
Population characteristics	For sighted participants: n = 8, 1 male and 7 females; Mean ± S.D. Age 30.75 ± 6.86, range 24-46 years.<br>For RP patients: n = 5, 3 males and 2 females; Mean ± S.D. Age 55.80 ± 12.40, range 40-71 years.
Recruitment	RP patients were recruited among those registered in the database of the clinics for inherited eye disease at the hospital. The principal investigator contacted patients by phone to suggest that they take part in the study. If the patient was interested, the appropriate informed consent was sent at home by post together with the updated invitation letter, so the participant had time (minimum 24 hr) to understand its content.<br>Sighted participants were recruited internally among the collaborators of the hospital. Sighted participants responding to a written notice by email were informed of the study and provided with the information-consent form at least 24h before their participation. Sighted participants were not directly or indirectly involved with the study nor its research question and had no hierarchical link to the study investigators.
Ethics oversight	Experiments with RP patients and SPs were conducted according to the Declaration of Helsinki and the ethical standards of the Swiss Federal Human Research Act (HRA). Experiments received approval from the Cantonal Commission for Ethics in Research (CER-VD). Approval ID 2023-01518 for RP patients and 2024-00999 for SPs. Informed consent was obtained from all human research participants.

Note that full information on the approval of the study protocol must also be provided in the manuscript.

Field-specific reporting

Please select the one below that is the best fit for your research. If you are not sure, read the appropriate sections before making your selection.

☒ Life sciences ☐ Behavioural & social sciences ☐ Ecological, evolutionary & environmental sciences

For a reference copy of the document with all sections, see nature.com/documents/nr-reporting-summary-flat.pdf

Life sciences study design

All studies must disclose on these points even when the disclosure is negative.

Sample size	No statistical method was used to predetermine the sample size. Sample size was chosen to minimize (3R) the number of mice in each group. In experiments animal groups were between 3 and 5 mice. Since each eye was demonstrated being an indipended unit, sample sizes in most experiment was twice the number of animals. Exceptions are in Fig. 4d,e,f and Supp Fig. 5a-c in which one eye is manipulated and the other is an internal control.
Data exclusions	No data were excluded from the analysis
Replication	Each experiment was replicated in multiple mice. The exact number of mice for each experiement is reported in each figure caption.
Randomization	Not relevant for the study.
Blinding	Blinding was not possible nor necessary. Experiments and data analysis were performed by the same person. Data obtained are from objective quantitative methods without any subjective bias.

Reporting for specific materials, systems and methods

We require information from authors about some types of materials, experimental systems and methods used in many studies. Here, indicate whether each material, system or method listed is relevant to your study. If you are not sure if a list item applies to your research, read the appropriate section before selecting a response.

Materials & experimental systems

n/a	Involved in the study
☒	☐ Antibodies
☒	☐ Eukaryotic cell lines
☒	☐ Palaeontology and archaeology
☐	☒ Animals and other organisms
☒	☐ Clinical data
☒	☐ Dual use research of concern
☒	☐ Plants

Methods

n/a	Involved in the study
☒	☐ ChIP-seq
☒	☐ Flow cytometry
☒	☐ MRI-based neuroimaging

Animals and other research organisms

Policy information about studies involving animals; ARRIVE guidelines recommended for reporting animal research, and Sex and Gender in Research

Laboratory animals	Experiments were performed in female C57BL/6J (control) mice (N = 13 mice, 27-70 years old), homozygous female C3H/HeNRj (RD1) mice (N = 36 mice, 29-198 years old), homozygous male/female B6.CXB1-Pde6brd10/J (RD10) mice (N = 5 mice, 101-260 years old), and homozygous male/female B6.129S6(Cg)-Rhotm1.1Kpal/J (P23H) mice (N = 4 mice, 140-146 years old). Control and RD1 mice were purchased from Janvier Labs (France). RD10 and P23H mice were bred in our facility.
Wild animals	The study did not involve wild animals
Reporting on sex	Both male and female mice were used. Sex is not a relevant factor in this study.
Field-collected samples	The study did not involve samples collected from the field
Ethics oversight	Animal experiments ex-vivo were performed according to Austrian laws governing animal experimentation. The research animal facility was approved by the responsible Federal Ministry of Education, Science and Research: BMWFW-66.009/0403-WF/V/3b/2014. Animal experiments in-vivo were conducted according to the animal authorisation VD3930 approved by the Direction générale de l'agriculture, de la viticulture et des affaires vétérinaires of the Canton de Vaud in Switzerland.

Note that full information on the approval of the study protocol must also be provided in the manuscript.

Plants

Seed stocks	*Report on the source of all seed stocks or other plant material used. If applicable, state the seed stock centre and catalogue number. If plant specimens were collected from the field, describe the collection location, date and sampling procedures.*
Novel plant genotypes	*Describe the methods by which all novel plant genotypes were produced. This includes those generated by transgenic approaches, gene editing, chemical/radiation-based mutagenesis and hybridization. For transgenic lines, describe the transformation method, the number of independent lines analyzed and the generation upon which experiments were performed. For gene-edited lines, describe the editor used, the endogenous sequence targeted for editing, the targeting guide RNA sequence (if applicable) and how the editor was applied.*
Authentication	*Describe any authentication procedures for each seed stock used or novel genotype generated. Describe any experiments used to assess the effect of a mutation and, where applicable, how potential secondary effects (e.g. second site T-DNA insertions, mosiacism, off-target gene editing) were examined.*
