# Supplementary material for: Resting-state electroretinography reveals pathological retinal oscillations in retinitis pigmentosa mice and patients
Source: Nat Commun. 2026 Jul 10;17:8537. doi: 10.1038/s41467-026-75520-9 (PMC13482292; doi:10.1038/s41467-026-75520-9)
Supplement: Supplementary file 4 [file 41467_2026_75520_MOESM5_ESM.pdf]

nature portfolio

Peer Review File

Resting-state electroretinography reveals pathological retinal oscillations in retinitis pigmentosa mice and patients

Corresponding Author: Dr Diego Ghezzi

This file contains all reviewer reports in order by version, followed by all author rebuttals in order by version.

Version 0:

Reviewer comments:

Reviewer #1

(Remarks to the Author)
This manuscript establishes a significant methodological advancement to record resting state ERG using flexible corneal electrodes and claims to demonstrate for the first time functional impacts of retinal remodeling triggered by photoreceptor death in patients. Moreover, it shows a pharmacological approach to address the negative impacts of retinal remodeling in multiple mouse models, a treatment that could be translated for vision restoration therapies for a broader patient population. Overall, this manuscript has significant clinical relevance, though, it does not provide significant new mechanistic insight. On the other hand, the finding that functional implications of retinal remodeling are also present in patients is highly significant. I have several comments, however, that will need to be addressed to strengthen confidence on the claims made specifically in human patients.

Major concerns:

One of the main weakness of this study is that the novel flexible corneal electrode array could not be used in patients. The results from alternative single-electrode method using multimetric analysis led to significantly less convincing results even in animal models (Figure 7b and c). Related issue is with title which indicates use of flexible corneal neurotechnology in both animal models and patients which is not the case. Finally, data shown in Fig 8 b shows very wide peak of oscillations both for controls and patients with relatively modest differences. There is significant overlap between control and patient data as shown in Fig 8c, reducing the ability of this method to diagnostically predict whether these oscillations are pathologic or not.

Second major concern is related to differences in anesthesia between Rd1 mice and controls. It appears that Rd1 mice are on different background than controls and there appears to be difference in breathing artefact between strains (and also between different ages, see Figure 3a). Since human data is without anesthesia and it is speculated that that may impact bursting activity, I am wondering if the difference in the observed oscillations could be due to differences in anesthesia level between different strains rather than photoreceptor degeneration. It could also be that GABA receptor modulation is impacting the depth of anesthesia since diazepam was applied systematically.

Minor concerns:

Introduction seems to suggest that AMD starts from photoreceptor degeneration, this might not be true.

Indicate clearly already in Results if recordings where done in darkness or in the presence of light (light level?).

Authors should comment why in Figure 3a there is a large variability in breathing artefact.

Overall, Results are heavy to read due to inclusion of all statistics in main text which disrupts the flow. Statistical analysis is important and overall done very well but it might be better to put details either in Figure legends and/or methods.

Local intravitreal injection of diazepam would be good addition to reduce the concerns of systemic origin of presented phenomenon and treatment effects.

Was experiment shown in Figure 6 done also in controls?

Discussion repeats Results a lot. Reducing review of results but on the other hand adding more discussion about potential mechanisms and alternative treatment options and future applications would make this article more interesting.

Reviewer #2

(Remarks to the Author)
In this manuscript, the authors present a non-invasive in vivo approach using a flexible corneal electrode combined with a dedicated analysis pipeline to investigate resting-state electroretinography. Using this technology, they demonstrate that retinal remodeling induces oscillatory activity in both RP mouse models and human patients. Overall, the study is interesting, and the authors provide solid experimental data along with a thorough analysis. Therefore, this work is suitable for Nature Communications after the authors address the following comments.
1. For Fig. 1f and 1g, the authors should specify the type of baseline used for the dual-peak Gaussian fitting. The authors should also provide fitting parameters (e.g., intensity, mean, FWHM) for the peak attributed to breathing.
2. In Fig. 2b, the RD1 mice show a gradual decrease in burst frequency over the recording, with dense bursts at the beginning that become sparser toward the end. What is the reason for this change?
3. In the RD1 mouse experiments (Fig. 1 and 2), the data collected from the left eye shows a higher mean and variance compared to those from the right eye. The authors should provide an explanation for this discrepancy.
4. In Fig. 3a, as the mice age from 4 to 7 weeks, an additional peak emerges between 20 and 25 Hz that cannot be overlooked. Is this peak also attributable to the second harmonic signal, as the authors later describe for the RD10 mice? The authors should provide an explanation here.
5. In Fig. 3a, the low-frequency peak is not observed in the PNW 6 mice. Why is this signal missing or significantly weaker compared to the corresponding peaks in the other groups?
6. For Fig. 3b-d, the author should provide R2 value for the linear regression not just for the gaussian fitting which could be misleading. Based on the linear regression results, it does not appear valid to claim a linear relationship between these three parameters and postnatal weeks. It may be better to describe the trends qualitatively.
7. For Fig. 5b, the author should add short description to explain why the measurements is carried to 2000 µA for RD1 group but not for the control group.
8. As the author mentioned, inhibitory amino acids can behave similarly as diazepam, then why were inhibitory amino acids not tested on mouse model as they potentially present less side effects and easier delivery methods?
Minor comments
9. Line 356, it should be Fig. 5f rather than 5e.
10. Line 463 should be Extended Data Figure 4 rather than Figure 3.
11. The format for references should be carefully reviewed to make sure it matches the requirements. The current format contains noticeable inconsistencies.

Reviewer #3

(Remarks to the Author)
I co-reviewed this manuscript with one of the reviewers who provided the listed reports. This is part of the Nature Communications initiative to facilitate training in peer review and to provide appropriate recognition for Early Career Researchers who co-review manuscripts.

Reviewer #4

(Remarks to the Author)
Summary
This study uses in-vivo resting-state electroretinograms (rsERG) to examine functional retinal remodeling in retinitis pigmentosa (RP). Using a flexible corneal neurotechnology combined with a sophisticated signal-processing pipeline, the authors demonstrate the presence of pathological oscillatory activity in multiple RP mouse models and, importantly, extend these findings to a small cohort of human patients. They further show that pharmacological suppression of oscillatory activity enhances retinal excitability to electrical stimulation.

Overall, the study addresses a significant gap in the field by moving well-established ex-vivo observations of retinal oscillations into an in-vivo context. Linking abnormal network dynamics to functional limitations is relevant for vision restoration strategies.

Major Comments
1) The authors emphasize the challenges of resting-state ocular recordings and implement a multi-metric artifact rejection and oscillation-validation pipeline. This is a strength of the paper. Nevertheless, because the human recordings are single-channel and lack independent monitoring of eye movements, blinks, EMG, or respiration, the possibility remains that residual artifacts could contribute to narrow-band detections. The authors should make a stronger case why the multi-criteria confirmation reduces false positives, and makes it unlikely that the detected oscillations are significantly impacted by subtle behavioral or physiological artifacts that are not directly measured remains a possibility. Providing examples of long, artifact-

stable epochs showing oscillatory activity versus matched control epochs might help strengthen confidence in their findings.
2) To reveal oscillatory activity in RD10 and P23H models, the authors modify anesthesia and employ paralysis with artificial ventilation to shift respiration-related noise outside the frequency band of interest. While this is methodologically reasonable and transparently discussed, it complicates cross-model comparisons and raises the possibility that protocol-dependent physiological differences contribute to the observed effects.
3) Comparing in vivo rsERG to previous ex vivo results: While detecting oscillatory activity of similar frequency as that measured ex vivo, suggest similar mechanisms may underlie them, the effects of Na blockers suggest otherwise. Previous ex vivo recording indicated that oscillations originate in the AII-CBC coupled network and were abolished by TTX. The authors should do a more thorough job of comparing results from previous studies and interpret their ERG data accordingly.
4) The demonstration that diazepam suppresses oscillations and enhances retinal excitability is an important functional result. However, because diazepam is administered systemically, it is difficult to attribute these effects specifically to retinal GABAA receptor modulation rather than central or peripheral effects. The link between retinal oscillations and improved stimulation efficacy would be strengthened by more targeted manipulations (e.g., local retinal benzo delivery/manipulation of known oscillation-generating mechanisms such as gap junctions).

Minor comments:
Since the use of the flexible MEA to do rsERG is one of the novel contributions to the field, perhaps some of these advantages can be brought up more directly instead of buried amongst the results.
lines 555-559: The authors refer to “dashed grey line in Fig. 7a” and “white dashed lines in Fig 7.a”, given the size of the panel, it might be helpful to label the lines so readers know exactly what to look for (otherwise they have to read back and realize that it is the line specifically in a particular numbered criterion box)
lines 627-628: “which are compatible with the highlight significant bands detected in pooled RP mouse models”. Should this be “highlighted”?
lines 675-677: “…retina which turns into efficiently driving V1 neurons” is awkward. Maybe “…retina, thus improving the output to V1 neurons” is more clear?
There isn’t a clear case made early on about why the multi-electrode array was better than the typical single electrode ERG setup on mice. Eventually it is mentioned that the presence of oscillations on multiple electrodes was used to filter out false positive events, a clear improvement on a single signal (which the authors note is similar to averaging the 8 electrodes together indicating that the area over which the electrodes are distributed is not necessarily meaningful). For example, at line 771 in the discussion, the single-channel nature of the human recordings makes it harder to throw out ocular artifacts.
Overall, this seems like a strong contribution to their field. That they were able to create a new non-invasive diagnostic tool using existing techniques simply by carefully designing a stringent analysis pipeline is very cool.

Gautam Awatramani and Geoff deRosenroll

Reviewer #5

(Remarks to the Author)
I co-reviewed this manuscript with one of the reviewers who provided the listed reports. This is part of the Nature Communications initiative to facilitate training in peer review and to provide appropriate recognition for Early Career Researchers who co-review manuscripts.

Version 1:

Reviewer comments:

Reviewer #1

(Remarks to the Author)
Authors addressed my original concerns comprehensively in their revision. It is significantly improved by new analysis that strenghtened conclusions based on patient data. They also addressed concerns about confounding impact of anesthesia. Local drug application, different blockers and expanded patient studies will be interesting future studies to better understand the impact of oscillations induced by photoreceptor degeneration.

Reviewer #2

(Remarks to the Author)
The authors have addressed all my comments with satisfaction.

Reviewer #3

(Remarks to the Author)
I co-reviewed this manuscript with one of the reviewers who provided the listed reports. This is part of the Nature Communications initiative to facilitate training in peer review and to provide appropriate recognition for Early Career Researchers who co-review manuscripts.

Reviewer #4

(Remarks to the Author)
The authors have done a good job of addressing the reviewers' concerns.

I have one remaining concern regarding the role of TTX-sensitive sodium channels in the generation of oscillations. Several previous studies, including Trenholm et al. (J Physiol. 2012;590:2501–2517; doi:10.1113/jphysiol.2011.225060), reported that TTX abolishes spontaneous oscillatory activity in the rd1 retina, suggesting an important role for voltage-gated sodium channels in generating or sustaining these rhythms. Given this prior literature, it would be helpful for the authors to cite these studies and discuss why their findings differ. Potential explanations could include differences in preparation, recording conditions, or the specific readout used to assess oscillatory activity.

Reviewer #5

(Remarks to the Author)
I co-reviewed this manuscript with one of the reviewers who provided the listed reports. This is part of the Nature Communications initiative to facilitate training in peer review and to provide appropriate recognition for Early Career Researchers who co-review manuscripts.

Open Access This Peer Review File is licensed under a Creative Commons Attribution 4.0 International License, which permits use, sharing, adaptation, distribution and reproduction in any medium or format, as long as you give appropriate credit to the original author(s) and the source, provide a link to the Creative Commons license, and indicate if changes were made.
In cases where reviewers are anonymous, credit should be given to 'Anonymous Referee' and the source.
The images or other third party material in this Peer Review File are included in the article's Creative Commons license, unless indicated otherwise in a credit line to the material. If material is not included in the article's Creative Commons license and your intended use is not permitted by statutory regulation or exceeds the permitted use, you will need to obtain permission directly from the copyright holder.
To view a copy of this license, visit https://creativecommons.org/licenses/by/4.0/

Reviewer #1 (Remarks to the Author):

This manuscript establishes a significant methodological advancement to record resting state ERG using flexible corneal electrodes and claims to demonstrate for the first time functional impacts of retinal remodeling triggered by photoreceptor death in patients. Moreover, it shows a pharmacological approach to address the negative impacts of retinal remodeling in multiple mouse models, a treatment that could be translated for vision restoration therapies for a broader patient population. Overall, this manuscript has significant clinical relevance, though, it does not provide significant new mechanistic insight. On the other hand, the finding that functional implications of retinal remodeling are also present in patients is highly significant. I have several comments, however, that will need to be addressed to strengthen confidence on the claims made specifically in human patients.
RE: we thank the reviewer for their positive comments and constructive suggestions, which are addressed point-by-point below. Motivated by their comment, we decided to investigate deeper the role of urethane providing new mechanistic insights into its effect on the retinal circuits and its related oscillation. These data are provided in Figure 6 and in Page 13 (Line 344) and a mechanistic explanation is provided at Page 23 (Line 591).

Major concerns:
One of the main weakness of this study is that the novel flexible corneal electrode array could not be used in patients. The results from alternative single-electrode method using multimetric analysis led to significantly less convincing results even in animal models (Figure 7b and c). Related issue is with title which indicates use of flexible corneal neurotechnology in both animal models and patients which is not the case. Finally, data shown in Fig 8 b shows very wide peak of oscillations both for controls and patients with relatively modest differences. There is significant overlap between control and patient data as shown in Fig 8c, reducing the ability of this method to diagnostically predict whether these oscillations are pathologic or not.
RE: the flexible corneal MEA is not approved for use in humans, and even if one were to substitute a comparable commercially available device that is approved for human neurocortical use (e.g., NeuroOne, WISE), it would still not be approved for ophthalmic application. For this reason, direct clinical translation of the corneal MEA approach is currently unrealistic, and we revised the text to ensure the manuscript does not imply otherwise.

Concerning the overall results in human subjects, we recognised that our original multi-metric approach, while effective in fully phenotyped conditions such as specific inbred mouse strains, was less efficient when data were pooled across groups, and did not fully exploit the richness of information available in time-frequency analysis. We also acknowledge that the single-channel nature of the human recordings limits artifact rejection through cross-electrode consistency. We therefore adopted a time-frequency approach based on scalogram analysis, applying blob detection — a method established in EEG pipelines — to identify and characterise oscillatory events. This yielded highly significant results in both mouse (Fig. 7) and human (Fig. 8) datasets, representing a substantial improvement over the previous approach, and we believe provides the objective and reproducible basis for confidence in the findings that the reviewer was seeking.

Here we summarise the key improvements of the new approach versus the previous approach.

1. Epoch-level rejection versus segment-level masking. The previous pipeline rejected entire 5-s epochs whenever rejection criteria were reached. While conservative, this strategy discarded large amounts of otherwise clean data when a single transient contaminant occurred within an epoch. Ultimately, this led to the exclusion of some eyes from patients and participants. The new analysis performs segment-level artifact masking rather than epoch-level rejection: the

precise time spans of detected artifacts are flagged and excluded from oscillation analysis, preserving the surrounding clean signal. This change simultaneously increases the yield of usable data and tightens the temporal specificity of artifact exclusion. All recorded eyes from patients and participants are now used.

2. Spectral cross-validation of artifacts using broadband columnar detection. A key insight motivating the revised pipeline was that virtually all artifacts detected by time-series criteria also produce a highly stereotyped signature in the corrected wavelet scalogram: a narrow-in-time, broadband-in-frequency columnar feature spanning at least 10 Hz. This spectral shape is mechanistically distinct from genuine oscillatory activity, which produces compact, frequency-limited blobs in the scalogram. By explicitly detecting and rejecting these columnar spectral structures, we now capture a class of artifacts — particularly those embedded within epochs with large baseline fluctuations — that the time-series z-score approach fails to flag. This two-domain cross-referencing (time-series thresholding plus spectral columnar detection) provides complementary coverage: transient-amplitude and derivative artifacts are caught by the time-series pass (Figure R1), while low-amplitude or baseline-shifted artifacts that nonetheless generate broadband spectral contamination are caught by the scalogram pass (Figure R2).

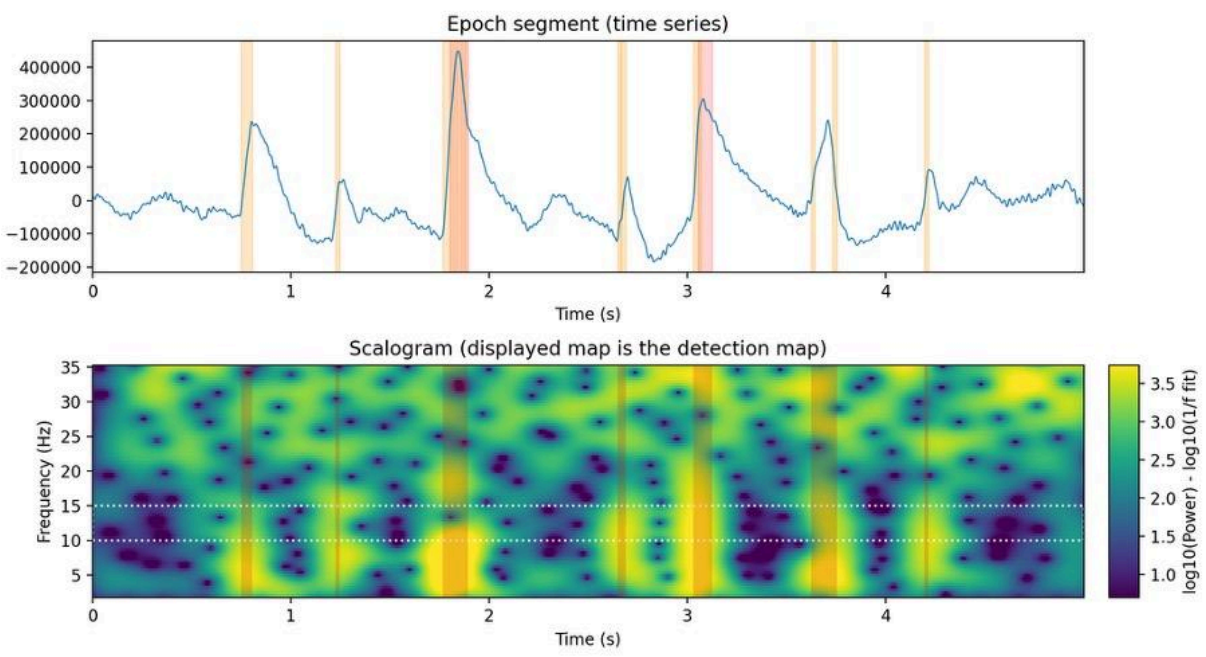

Figure R1. Time-series and spectral artifact detection are complementary. Artifact segments identified by the derivative criterion (orange) and amplitude criterion (pink) are overlaid on the corrected wavelet scalogram. Each artifact produces a corresponding narrow-in-time, broadband columnar signature in the scalogram, spanning more than 10 Hz, consistent with non-oscillatory transient activity.

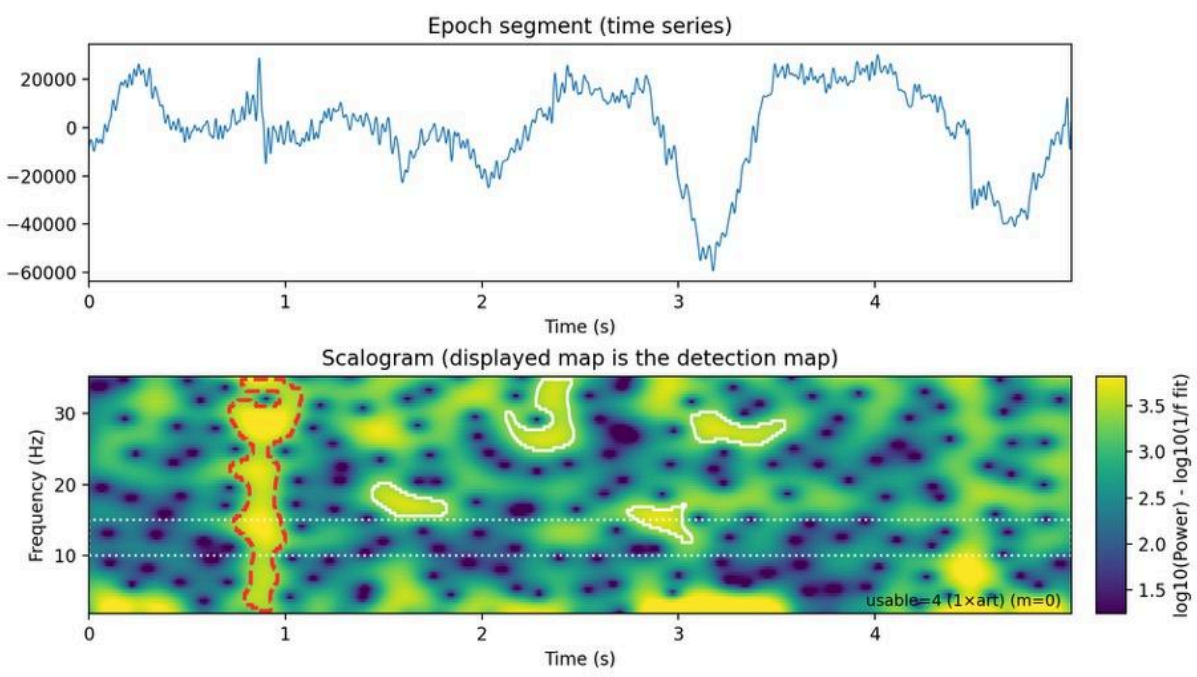

Figure R2. Wavelet-based columnar detection captures artifacts missed by time-series z-scoring. In this epoch, large baseline fluctuations suppress the robust z-score of an embedded artifact below threshold, preventing its detection by the timeseries criterion alone. The same artifact is unambiguously identified by its characteristic broadband columnar signature in the corrected scalogram.

3. Replacing fixed-band filtering with data-driven blob-defined bandpass filtering. The previous pipeline applied sequential narrow 3 Hz bandpass filters across the frequency range and then searched each filtered signal for elevated Hilbert envelope, wavelet ridge power, and phase autocorrelation. This approach carries two artifact-related risks. First, narrow filtering can impose pseudo-regularity on broadband noise, inflating cycle-by-cycle consistency metrics. Second, a genuine oscillation spanning a band larger than 3 Hz would be artificially fragmented across multiple filter bands, reducing detection power while simultaneously risking false positives at band edges. In the revised pipeline, we first detect and characterise wavelet blobs directly in the corrected scalogram, then use each blob's empirically determined frequency span to define the bandpass filter for subsequent cycle-by-cycle analysis. Because the bandpass is derived from a region where oscillatory power has already been confirmed in the

time-frequency domain — and because broadband columnar artifacts are explicitly excluded at the scalogram stage — narrowband filter-induced pseudo-oscillations are strongly suppressed. Critically, this approach also provides an independent spectral criterion against which every candidate burst is implicitly validated: a detection can only arise from a frequency-limited blob, never from a broadband spectral region consistent with artifact.

Second major concern is related to differences in anesthesia between Rd1 mice and controls. It appears that Rd1 mice are on different background than controls and there appears to be difference in breathing artefact between strains (and also between different ages, see Figure 3a). Since human data is without anesthesia and it is speculated that that may impact bursting activity, I am wondering if the difference in the observed oscillations could be due to differences in anesthesia level between different strains rather than photoreceptor degeneration. It could also be that GABA receptor modulation is impacting the depth of anesthesia since diazepam was applied systematically.
RE: in mice data (compared to humans) the most important role of anesthesia is to keep mice calm and minimize movement artifacts. This is not a concern in participants since they can remain at rest voluntarily. Specifically for the mouse experiments, in the revised manuscript we performed additional analysis to verify that changes in the high-frequency peak (oscillatory activity) are not driven by systemic changes in anesthetic depth.

First, the new analysis demonstrates that the low-frequency PSD component associated with breathing is highly variable (as noted by the reviewer), because it depends on the physiological breathing dynamics. This variability occurs not only between strains, but also within the same strain, within the same animal over the course of anesthesia, and even between microelectrodes of the same array. This point is shown in Supplementary Figure 2 and clarified in Page 3 (Line 81). Second, the analysis shows that the low-frequency PSD component related to breathing does not correlate to the power amplitude at the high-frequency (oscillatory activity) as reported in Supplementary Figure 2d and in Page 3 (Line 90). With this analysis we clarify that the change in spectral power related to breathing does not influence the high-frequency peak (oscillatory activity). Therefore, we confirm that changes in the high-frequency peak (oscillatory activity) are not driven by systemic changes in anesthetic depth.

In addition, we verified that the reduction in the high-frequency peak (oscillatory activity) following diazepam administration is not correlated with changes in PSD power at low-frequency (breathing artifact). This result is reported in Supplementary Figure 4. Specifically, the high-frequency peak (oscillatory activity) always decreases, while the breathing artifact may decrease, increase or remain stable across the 10 recorded eyes. These two measures are not correlated.

Overall, our new analysis indicates that, although depth of anesthesia may vary between strains, ages, and experimental conditions, variation in the low-frequency artifact does not correlate with the high-frequency PSD peak (oscillatory activity).

Minor concerns:
Introduction seems to suggest that AMD starts from photoreceptor degeneration, this might not be true.
RE: we modified the sentence and limited introduction to RP.

Indicate clearly already in Results if recordings where done in darkness or in the presence of light (light level?).
RE: we now indicated a sentence at various points of the methods section, as follows “Mice were not dark-adapted, but kept in dark within an aluminium enclosure (XE25C11D, Thorlabs) during recordings”.

Authors should comment why in Figure 3a there is a large variability in breathing artefact.
RE: as indicated above, we have included a new Supplementary Figure 2 showing that the breathing component of the PSD is variable, even within the same eye over time. Essentially, breathing is a physiological process (not a stable clock) that changes depending on multiple phenomena. It is not surprising then that it varies from recordings that are one week apart.

Overall, Results are heavy to read due to inclusion of all statistics in main text which disrupts the flow. Statistical analysis is important and overall done very well but it might be better to put details either in Figure legends and/or methods.
RE: this is a usual problem. We tried our best to place them into figure captions. However, figure legends are limited to 350 words. Therefore, lengthy statistical results have been moved to Supplementary Notes. We hope this would be acceptable.

Local intravitreal injection of diazepam would be good addition to reduce the concerns of systemic origin of presented phenomenon and treatment effects.
RE: we appreciate this suggestion and attempted to address it. Diazepam itself is not suitable for local intravitreal delivery due to its formulation and solubility constraints, so we instead performed intravitreal injections of GABA-A and GABA-B modulators (THIP and baclofen, respectively) as an alternative approach – given evidence of their impact impact on light-evoked ERG signals following IVT injection (see J. Wang et al., Experimental Eye Research 139 (2015) 48e63).

While initial results appeared promising, we consistently observed significant changes in retinal oscillatory activity in the fellow eye receiving vehicle (saline) injection alone. This bilateral effect indicates that the injection procedure itself was sufficiently traumatic to confound interpretation, independently of any pharmacological action. Given that this contralateral disturbance could not be reliably controlled, we concluded that intravitreal injection in our hands does not currently provide the clean experimental conditions required to draw meaningful conclusions about local versus systemic drug action and have therefore not included these data in the present study.

Was experiment shown in Figure 6 done also in controls?
RE: we did not perform mechanical ventilation and paralysis in control mice. However, several other control experiments were performed to directly address the potential contribution of breathing- or movement-related artifacts to the observed oscillatory activity. First, WT mice recorded under ketamine/xylazine anesthesia never exhibited the pathological 10–15 Hz oscillatory activity observed in RD1 mice (Figure 1). Second, RD1 mice recorded under ketamine/xylazine together with mechanical ventilation and paralysis still exhibited the same oscillatory activity, demonstrating that ventilation/paralysis itself does not generate or account for the oscillatory phenotype (Figure 6). Third, when RD mice were recorded under urethane together with ventilation/paralysis (Figure 6), the oscillatory peak remained present but shifted slightly in frequency, indicating that urethane modulates intrinsic retinal network activity rather than revealing an artifact related to respiration or movement.

In addition, the new analyses included in the revised manuscript (Supplementary Figure 2 and 4) further demonstrate that the low-frequency breathing-related PSD component does not correlate

with the high-frequency oscillatory peak across strains, ages, anesthetic conditions, or diazepam experiments. Collectively, these results indicate that the pathological oscillatory activity is intrinsic to the degenerating retina and is not explained by breathing-related artifacts, movement, or the use of ventilation/paralysis.

Given these convergent control experiments and analyses, we did not consider it scientifically necessary to systematically extend mechanical ventilation and paralysis to all WT control conditions. Moreover, these procedures are highly invasive in mice and were therefore limited to the experiments necessary to answer questions that could not be answered with alternative experiments.

Discussion repeats Results a lot. Reducing review of results but on the other hand adding more discussion about potential mechanisms and alternative treatment options and future applications would make this article more interesting.
RE: we modified the discussion as indicated.

Reviewer #2 (Remarks to the Author):

In this manuscript, the authors present a non-invasive in vivo approach using a flexible corneal electrode combined with a dedicated analysis pipeline to investigate resting-state electroretinography. Using this technology, they demonstrate that retinal remodeling induces oscillatory activity in both RP mouse models and human patients. Overall, the study is interesting, and the authors provide solid experimental data along with a thorough analysis. Therefore, this work is suitable for Nature Communications after the authors address the following comments.
RE: we thank the reviewer for their positive comments and constructive suggestions, which are addressed point-by-point below.

1. For Fig. 1f and 1g, the authors should specify the type of baseline used for the dual-peak Gaussian fitting. The authors should also provide fitting parameters (e.g., intensity, mean, FWHM) for the peak attributed to breathing.
RE: the baseline (knee mode) is now reported in the text (methods: "Spectral analysis of resting-state ERG"). As we explain in the text (Page 5) the fitting parameters have been optimised to provide best fitting for the peak corresponding to neural oscillation (which is the scope of the paper). Also, the FOOOF algorithm (or any gaussian fitting algorithm) might encounter problems to provide good fitting in the low-frequency zone close to the cutoff frequency of the band-pass filter (1 Hz), due to the border effect. These are the reasons why we do not provide data for the first peak. We mentioned that it can be fitted, and it is true, but the fitting quality is not as reliable as the other peak.

2. In Fig. 2b, the RD1 mice show a gradual decrease in burst frequency over the recording, with dense bursts at the beginning that become sparser toward the end. What is the reason for this change?
RE: The raster is an illustrative random example showing a short 100s excerpt from a 3600s (60 min) recording. Therefore, it is not intended to imply a systematic short-term decrease in burst rate. In the revision, we clarified this in the figure caption.

3. In the RD1 mouse experiments (Fig. 1 and 2), the data collected from the left eye shows a higher mean and variance compared to those from the right eye. The authors should provide an explanation for this discrepancy.
RE: it is true that the plots show a higher mean for the LE (but this is just the mean). Indeed eyes are different, and it is not realistic to expect exactly the same value in both eyes. However, this difference is not significant. Moreover, LE is not consistently higher on a per mouse base. For example, in Figure 1e RD1, LE is higher in 2 mice and RE is higher in the other two mice. Paired values are below (the higher for each mouse is in bold).

**RD1 figure 1e**	**Left Eye**	**Right Eye**
Mouse 1	17.89436	**24.69864**
Mouse 2	11.59939	**13.50504**
Mouse 3	**30.52424**	21.28832
Mouse 4	**19.1985**	12.03202

4. In Fig. 3a, as the mice age from 4 to 7 weeks, an additional peak emerges between 20 and 25 Hz that cannot be overlooked. Is this peak also attributable to the second harmonic signal, as the authors later describe for the RD10 mice? The authors should provide an explanation here.
RE: a peak at higher frequencies (20-25 Hz) emerges sometimes. Providing explanations about this peak is difficult. Indeed, in Supplementary Figure 8 it is shown that a peak around this frequency is visible in multiple mice models including controls. Being visible in controls, it is difficult to assign this peak to a harmonic of a signal. We made the hypothesis (Page 18, Line 457) that this peak is associated with the spontaneous rhythm observed in RGC at constant light level (on in dark) described by Neuenschwander in cat retinas [Neuenschwander, S., Castelo-Branco, M. & Singer, W. Synchronous oscillations in the cat retina. Vis. Res. 39, 2485–2497 (1999).].

5. In Fig. 3a, the low-frequency peak is not observed in the PNW 6 mice. Why is this signal missing or significantly weaker compared to the corresponding peaks in the other groups?
RE: the low-frequency component related to breathing in PSD is variable since it depends on the physiological breathing dynamics. It is not only variable between strains, but also within the same strain, within the same animal along anaesthesia time, and sometimes also between microelectrodes of the same array. This point is shown in Supplementary Figure 2. Essentially, breathing is a physiological process (not a stable clock) that changes depending on multiple phenomena. It is not surprising then that it varies from recordings that are one week apart.

6. For Fig. 3b-d, the author should provide R2 value for the linear regression not just for the gaussian fitting which could be misleading. Based on the linear regression results, it does not appear valid to claim a linear relationship between these three parameters and postnatal weeks. It may be better to describe the trends qualitatively.
RE: correct, we have forgotten them. They are now indicated in the legend of Figure 2.

7. For Fig. 5b, the author should add short description to explain why the measurements is carried to 2000 μA for RD1 group but not for the control group.
RE: in controls EEP amplitudes saturates at lower currents. Therefore, we decided to not stimulate at very high currents to avoid risk of stimulation induced pain since it is not necessary. This explanation has been included at Page 9 (Line 231).

8. As the author mentioned, inhibitory amino acids can behave similarly as diazepam, then why were inhibitory amino acids not tested on mouse model as they potentially present less side effects and easier delivery methods?
RE: we based our choice on previous literature. Benzodiazepines were already tested in ex-vivo experiment with explanted retinas. Hence, we expected to observe an effect to prove the validity of the recording methods. Inhibitory amino acids is a hypothesis that we will test in future studies.

Minor comments
9. Line 356, it should be Fig. 5f rather than 5e.
10. Line 463 should be Extended Data Figure 4 rather than Figure 3.
11. The format for references should be carefully reviewed to make sure it matches the requirements. The current format contains noticeable inconsistencies.
RE: all minor comments were addressed.

Reviewer #4 (Remarks to the Author):

Summary

This study uses in-vivo resting-state electroretinograms (rsERG) to examine functional retinal remodeling in retinitis pigmentosa (RP). Using a flexible corneal neurotechnology combined with a sophisticated signal-processing pipeline, the authors demonstrate the presence of pathological oscillatory activity in multiple RP mouse models and, importantly, extend these findings to a small cohort of human patients. They further show that pharmacological suppression of oscillatory activity enhances retinal excitability to electrical stimulation.

Overall, the study addresses a significant gap in the field by moving well-established ex-vivo observations of retinal oscillations into an in-vivo context. Linking abnormal network dynamics to functional limitations is relevant for vision restoration strategies.

RE: we thank the reviewer for their positive comments and constructive suggestions, which are addressed point-by-point below.

Major Comments

1) The authors emphasize the challenges of resting-state ocular recordings and implement a multi-metric artifact rejection and oscillation-validation pipeline. This is a strength of the paper. Nevertheless, because the human recordings are single-channel and lack independent monitoring of eye movements, blinks, EMG, or respiration, the possibility remains that residual artifacts could contribute to narrow-band detections. The authors should make a stronger case why the multi-criteria confirmation reduces false positives, and makes it unlikely that the detected oscillations are significantly impacted by subtle behavioral or physiological artifacts that are not directly measured remains a possibility. Providing examples of long, artifact-stable epochs showing oscillatory activity versus matched control epochs might help strengthen confidence in their findings.

RE: we thank the reviewer for this suggestion. While we considered adding representative traces, we were concerned that only selecting visually clean segments — however representative — could itself be perceived as confirmatory bias, particularly given that artifact susceptibility is central to the concern being raised. While examples of bursting oscillatory activity are now reported in Figure 8c (similar to examples reported for mice in Fig. 7), we reasoned that a quantitative improvement to the analysis would more rigorously address the underlying question of result reliability than trace selection alone. We also believe that a rigorous analysis pipeline is a key contribution to the field allowing robust results also when clinically-validated single-channel ERG electrodes are used without independent monitoring of eye movements, blinks, or EMG.

To this end, we recognised that our original multi-metric approach, while effective in fully phenotyped conditions such as specific inbred mouse strains, was less efficient when data were pooled across groups, and did not fully exploit the richness of information available in time-frequency analysis. We also acknowledge that the single-channel nature of the human recordings limits artifact rejection through cross-electrode consistency. We therefore adopted a time-frequency approach based on scalogram analysis, applying blob detection — a method established in EEG pipelines — to identify and characterise oscillatory events. This yielded highly significant results in both mouse (Fig. 7) and human (Fig. 8) datasets, representing a substantial improvement over the previous approach, and we believe provides the objective and reproducible basis for confidence in the findings that the reviewer was seeking.

Here we summarise the key improvements of the new approach versus the previous approach.

1. Epoch-level rejection versus segment-level masking. The previous pipeline rejected entire 5-s epochs whenever rejection criteria were reached. While conservative, this strategy discarded

large amounts of otherwise clean data when a single transient contaminant occurred within an epoch. Ultimately, this led to the exclusion of some eyes from patients and participants. The new analysis performs segment-level artifact masking rather than epoch-level rejection: the precise time spans of detected artifacts are flagged and excluded from oscillation analysis, preserving the surrounding clean signal. This change simultaneously increases the yield of usable data and tightens the temporal specificity of artifact exclusion. All recorded eyes from patients and participants are now used.

2. Spectral cross-validation of artifacts using broadband columnar detection. A key insight motivating the revised pipeline was that virtually all artifacts detected by time-series criteria also produce a highly stereotyped signature in the corrected wavelet scalogram: a narrow-in-time, broadband-in-frequency columnar feature spanning at least 10 Hz. This spectral shape is mechanistically distinct from genuine oscillatory activity, which produces compact, frequency-limited blobs in the scalogram. By explicitly detecting and rejecting these columnar spectral structures, we now capture a class of artifacts — particularly those embedded within epochs with large baseline fluctuations — that the time-series z-score approach fails to flag. This two-domain cross-referencing (time-series thresholding plus spectral columnar detection) provides complementary coverage: transient-amplitude and derivative artifacts are caught by the time-series pass (Figure R1), while low-amplitude or baseline-shifted artifacts that nonetheless generate broadband spectral contamination are caught by the scalogram pass (Figure R2).

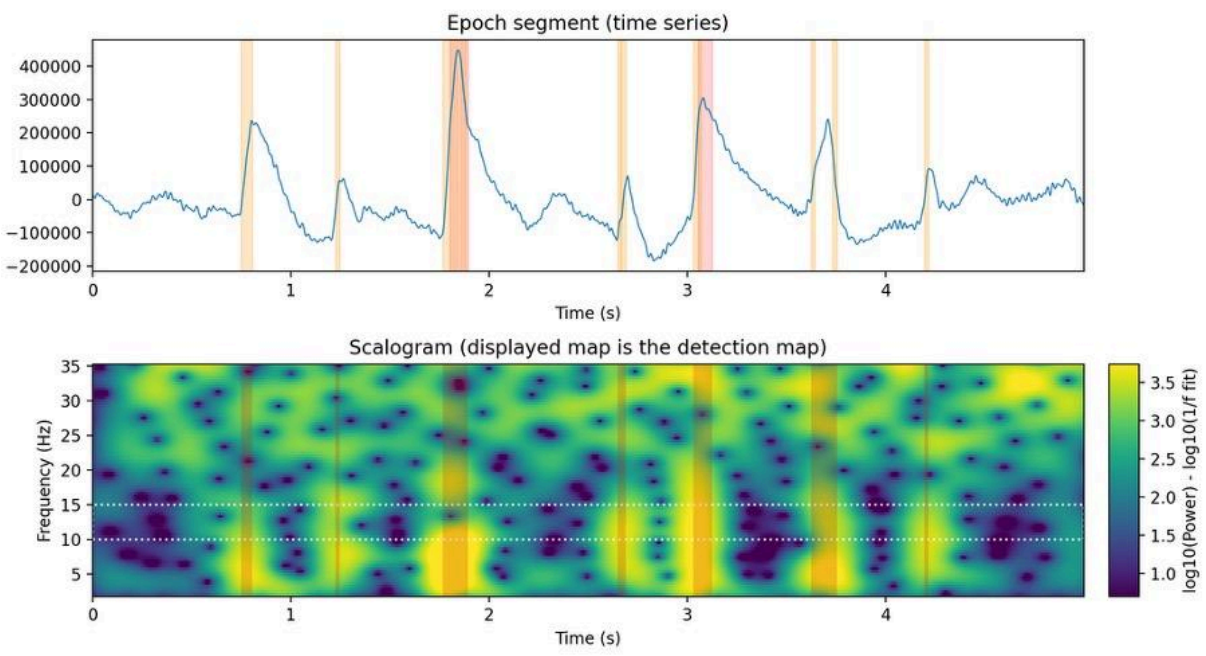

Figure R1. Time-series and spectral artifact detection are complementary. Artifact segments identified by the derivative criterion (orange) and amplitude criterion (pink) are overlaid on the corrected wavelet scalogram. Each artifact produces a corresponding narrow-in-time, broadband columnar signature in the scalogram, spanning more than 10 Hz, consistent with non-oscillatory transient activity.

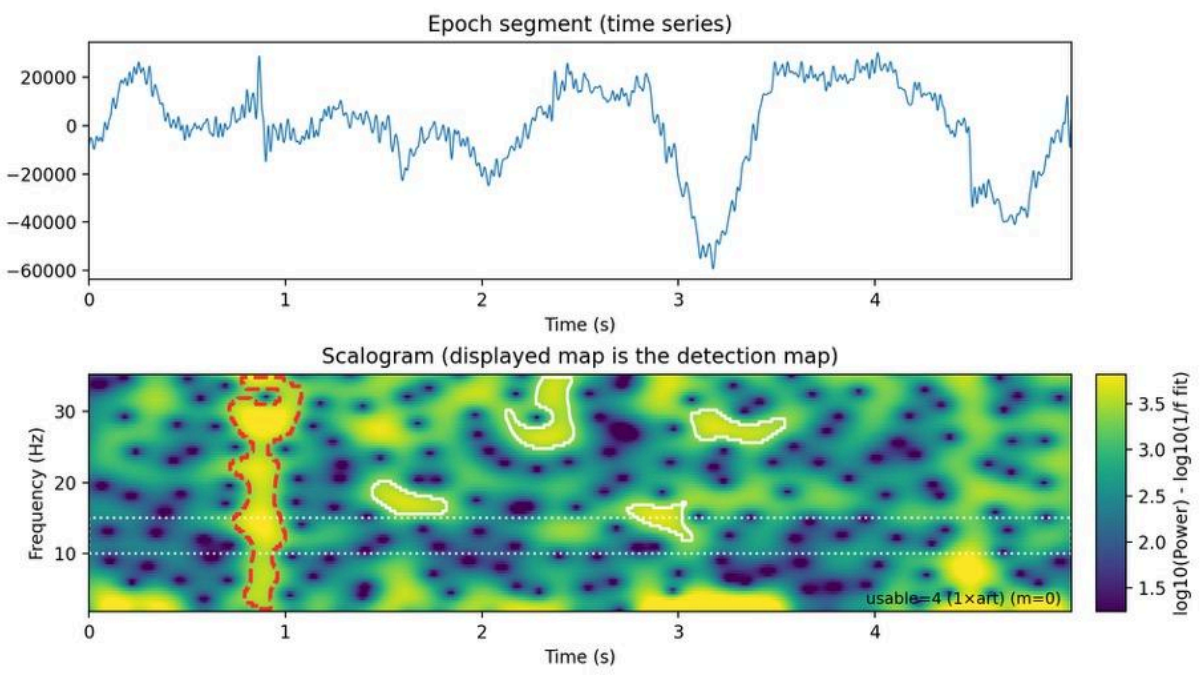

Figure R2. Wavelet-based columnar detection captures artifacts missed by time-series z-scoring. In this epoch, large baseline fluctuations suppress the robust z-score of an embedded artifact below threshold, preventing its detection by the timeseries criterion alone. The same artifact is unambiguously identified by its characteristic broadband columnar signature in the corrected scalogram.

3. Replacing fixed-band filtering with data-driven blob-defined bandpass filtering. The previous pipeline applied sequential narrow 3 Hz bandpass filters across the frequency range and then searched each filtered signal for elevated Hilbert envelope, wavelet ridge power, and phase autocorrelation. This approach carries two artifact-related risks. First, narrow filtering can impose pseudo-regularity on broadband noise, inflating cycle-by-cycle consistency metrics. Second, a genuine oscillation spanning a band larger than 3 Hz would be artificially fragmented across multiple filter bands, reducing detection power while simultaneously risking false positives at band edges. In the revised pipeline, we first detect and characterise wavelet blobs

directly in the corrected scalogram, then use each blob's empirically determined frequency span to define the bandpass filter for subsequent cycle-by-cycle analysis. Because the bandpass is derived from a region where oscillatory power has already been confirmed in the time-frequency domain — and because broadband columnar artifacts are explicitly excluded at the scalogram stage — narrowband filter-induced pseudo-oscillations are strongly suppressed. Critically, this approach also provides an independent spectral criterion against which every candidate burst is implicitly validated: a detection can only arise from a frequency-limited blob, never from a broadband spectral region consistent with artifact.

2) To reveal oscillatory activity in RD10 and P23H models, the authors modify anesthesia and employ paralysis with artificial ventilation to shift respiration-related noise outside the frequency band of interest. While this is methodologically reasonable and transparently discussed, it complicates cross-model comparisons and raises the possibility that protocol-dependent physiological differences contribute to the observed effects.
RE: in RD10 and P23H we only provide evidence that the oscillation is present but at a different dominant frequency compared to RD1 (respectively ~5 Hz vs ~10 Hz). While it is reasonable to ask if this difference is due to protocol differences, we also clarified that ex-vivo literature already reported 5 Hz as dominant frequency for RD10, while our own ex-vivo data shows that P23H retinas also have a 5 Hz dominant frequency. Ex-vivo experiments on retinal explants serve as ground-truth since the variables are controlled. Nevertheless, in the revised paper we removed as possible cross-model comparison especially when data from multimetric analysis were pooled together.

More importantly, we decided to investigate deeper if urethane would have an effect. Toward this goal we provide new in RD1 comparing urethane vs KX anesthesia under mechanical ventilation and paralysis (Fig. 6). Here we found that oscillatory activity is altered by urethane specifically (a notion that was already reported in the original paper from Dräger and Hubel. In page 23 (Line 591) of the discussion we provide a putative explanation of the effect of urethane.

3) Comparing in vivo rsERG to previous ex vivo results: While detecting oscillatory activity of similar frequency as that measured ex vivo, suggest similar mechanisms may underlie them, the effects of Na blockers suggest otherwise. Previous ex vivo recording indicated that oscillations originate in the AII-CBC coupled network and were abolished by TTX. The authors should do a more thorough job of comparing results from previous studies and interpret their ERG data accordingly.
RE: the seminal paper in RD1 retinas from Mezler&Zeck (10.1523/jneurosci.4238-10.2011) shows that TTX effectively abolishes RGC spikes but does not inhibit neuronal oscillation (their figure 5). Resting-state ERG is unlikely to record neuronal spikes (due to the distance from the electrode to the retina) but it is measuring neuronal oscillations. Therefore the in-vivo and ex-vivo effects of sodium channel blockers (Oxy and TTX respectively) are in our opinion coherent. We clarified this point in the manuscript in Page 8 (Line 201) and Page 9 (Line 216).

4) The demonstration that diazepam suppresses oscillations and enhances retinal excitability is an important functional result. However, because diazepam is administered systemically, it is difficult to attribute these effects specifically to retinal GABAA receptor modulation rather than central or peripheral effects. The link between retinal oscillations and improved stimulation efficacy would be strengthened by more targeted manipulations (e.g., local retinal benzo delivery/manipulation of known oscillation-generating mechanisms such as gap junctions).
RE: we agree with this comment and attempted to address it. Diazepam itself is not suitable for local intravitreal delivery due to its formulation and solubility constraints, so we instead performed

intravitreal injections of GABA-A and GABA-B modulators (THIP and baclofen, respectively) as an alternative approach – given evidence of their impact impact on light-evoked ERG signals following IVT injection (see J. Wang et al., Experimental Eye Research 139 (2015) 48e63).

While initial results appeared promising, we consistently observed significant changes in retinal oscillatory activity in the fellow eye receiving vehicle (saline) injection alone. This bilateral effect indicates that the injection procedure itself was sufficiently traumatic to confound interpretation, independently of any pharmacological action. Given that this contralateral disturbance could not be reliably controlled, we concluded that intravitreal injection in our hands does not currently provide the clean experimental conditions required to draw meaningful conclusions about local versus systemic drug action and have therefore not included these data in the present study.

Nevertheless, to circumvent the mentioned problem, there are two experiments in the paper. Concerning transcorneal stimulation, diazepam is given to control mice as well (Figure 4e). While absolute amplitudes are reduced (Supplementary Figure 6c), sigmoidal fitting and EC50 are not altered. If the effect observed in RD1 is caused by systemic effect, this would have also occurred in control mice. In addition, we added new rsERG analysis (Supplementary Figure 4). Diazepam influences breathing behaviour in mice (systemic effect) but the neuronal oscillatory activity reduction observed is not correlated to the change in PSD power at low-frequency (breathing artefact). Essentially, the oscillatory activity always decreases, while the breathing artifact decreases, increases or remains stable across the 10 measured eyes. The two variations are not correlated. These two results reinforce the idea that the effect is retinal and not systemic.

Minor comments:
Since the use of the flexible MEA to do rsERG is one of the novel contributions to the field, perhaps some of these advantages can be brought up more directly instead of buried amongst the results.
RE: yes.

lines 555-559: The authors refer to “dashed grey line in Fig. 7a” and “white dashed lines in Fig 7.a”, given the size of the panel, it might be helpful to label the lines so readers know exactly what to look for (otherwise they have to read back and realize that it is the line specifically in a particular numbered criterion box)
RE: this figure has been completely changed.

lines 627-628: “which are compatible with the highlight significant bands detected in pooled RP mouse models”. Should this be “highlighted”?
RE: this is not anymore in the paper.

lines 675-677: “…retina which turns into efficiently driving V1 neurons” is awkward. Maybe “…retina, thus improving the output to V1 neurons” is more clear?
RE: yes, the sentence was changed.

There isn’t a clear case made early on about why the multi-electrode array was better than the typical single electrode ERG setup on mice. Eventually it is mentioned that the presence of oscillations on multiple electrodes was used to filter out false positive events, a clear improvement on a single signal (which the authors note is similar to averaging the 8 electrodes together indicating that the area over which the electrodes are distributed is not necessarily meaningful). For example, at line 771 in the discussion, the single-channel nature of the human recordings makes it harder to throw out ocular artifacts. Overall, this seems like a strong contribution to their

field. That they were able to create a new non-invasive diagnostic tool using existing techniques simply by carefully designing a stringent analysis pipeline is very cool.
RE: In mice, the most important advantage is given by using a thin flexible array that is firmly attached to the cornea, partially dampening large movements, with both recording and ground electrodes on the same array. Conventional ERG settings have the active electrode on the cornea and the return electrode on the skin. This is not ideal as well. Improvements are mentioned at the beginning of the results section in Page 3 (Line 58).

Mice data were much cleaner with almost no artefacts. Therefore, we did not take advantage of having multiple active electrodes to unambiguously flag artifacts. On the contrary, such an array would have been extremely useful in human data to flag artifacts in a simpler manner. This is why we had to put in place a stringent analysis technique. We clarified this point in the discussion at Page 25 (Line 633).

Gautam Awatramani and Geoff deRosenroll

Reviewer #3 (Remarks to the Author):

I co-reviewed this manuscript with one of the reviewers who provided the listed reports. This is part of the Nature Communications initiative to facilitate training in peer review and to provide appropriate recognition for Early Career Researchers who co-review manuscripts.
RE: we thank the reviewer for their positive comments and constructive suggestions, which are addressed point-by-point.

Reviewer #5 (Remarks to the Author):

I co-reviewed this manuscript with one of the reviewers who provided the listed reports. This is part of the Nature Communications initiative to facilitate training in peer review and to provide appropriate recognition for Early Career Researchers who co-review manuscripts.
RE: we thank the reviewer for their positive comments and constructive suggestions, which are addressed point-by-point.

Reviewer #1 (Remarks to the Author):
Authors addressed my original concerns comprehensively in their revision. It is significantly improved by new analysis that strenghtened conclusions based on patient data. They also addressed concerns about confounding impact of anesthesia. Local drug application, different blockers and expanded patient studies will be interesting future studies to better understand the impact of oscillations induced by photoreceptor degeneration.
RE: we thank the reviewer for their positive assessment.

Reviewer #2 (Remarks to the Author):
The authors have addressed all my comments with satisfaction.
RE: we thank the reviewer for their positive assessment.

Reviewer #3 (Remarks to the Author):
I co-reviewed this manuscript with one of the reviewers who provided the listed reports. This is part of the Nature Communications initiative to facilitate training in peer review and to provide appropriate recognition for Early Career Researchers who co-review manuscripts.
RE: we thank the reviewer for their positive assessment.

Reviewer #4 (Remarks to the Author):
The authors have done a good job of addressing the reviewers' concerns.
I have one remaining concern regarding the role of TTX-sensitive sodium channels in the generation of oscillations. Several previous studies, including Trenholm et al. (J Physiol. 2012;590:2501–2517; doi:10.1113/jphysiol.2011.225060), reported that TTX abolishes spontaneous oscillatory activity in the rd1 retina, suggesting an important role for voltage-gated sodium channels in generating or sustaining these rhythms. Given this prior literature, it would be helpful for the authors to cite these studies and discuss why their findings differ. Potential explanations could include differences in preparation, recording conditions, or the specific readout used to assess oscillatory activity.
RE: we thank the reviewer for their positive assessment. We sincerely thank the reviewer for insisting on this point and pointing us to the reference. We have updated our text to include these additional observations. In the end, since eye drops are designed to act on the eye surface and not reach the retina in a therapeutically meaningful concentration, literature results can explain our data considering the low dose effectively reaching the retina. From our point of view, what is important is that a conventionally used anesthetic in ERG does not alter detection capability.
Here the modified text:

Finally, prior ex-vivo experiments in RD1 explanted retinas showed contrasting evidence on the role of voltage-gated sodium channels in maintaining oscillatory activity. One study showed that blocking voltage-gated sodium channels with tetrodotoxin abolishes RGC spiking activity without reducing neuronal oscillations in RGCs[15], while another showed that both spiking and oscillatory activities are reduced in RGCs[19]. A possible explanation for this discrepancy is the different dose used in the two studies: a lower dose (0.2 μM) in the first one and a higher dose in the second one (1 μM). Oxybuprocaine is a local anesthetic binding voltage-gated sodium channels routinely used in ophthalmology, including during light-evoked ERG. Therefore, we tested whether the topical administration of oxybuprocaine (0.4% eye drops) alters in-vivo rsERG. We recorded rsERG in five RD1 mice (49-51 days old) for 30 min before and 30 min after topical administration in one eye only, with a 10 min gap between recordings, using the untreated eye as an internal control

(**Supplementary Fig. 5**). Peak frequency and log peak amplitude remained unchanged in both the treated and untreated eyes. Only, the FWHM showed a slight decrease in both groups (results of two-way repeated measures ANOVA and Šídák's multiple comparisons tests in **Supplementary Fig. 5**). The mean goodness of fit is $R^2 = 0.99 \pm 0.01$ before, $R^2 = 0.99 \pm 0.01$ in the untreated eyes and $R^2 = 0.99 \pm 0.01$ in the treated eyes (± S.D.).

Although the systemic administration of diazepam precludes definitive attribution of the observed effects specifically to retinal $GABA_A$ receptors and does not fully exclude extra-retinal contributions, these data together with previous ex-vivo findings demonstrate that retinal oscillatory activity is markedly suppressed in-vivo by a positive allosteric modulator of $GABA_A$ receptors. This result has translational relevance, as previous studies in explanted mouse retinas have shown that suppressing bursting oscillatory activity improves the efficacy of both electrical and optogenetic retinal stimulation[25,36]. In addition, the commonly used local anesthetic oxybuprocaine did not alter rsERG detection capability. Since oxybuprocaine eye drops are not designed to reach the retina at a therapeutically meaningful concentration, this result is consistent with the previous ex vivo observation in RD1 explanted retinas showing that a low dose of tetrodotoxin does not diminish retinal oscillatory activity[15]. This result further supports the clinical translatability of rsERG since oxybuprocaine is frequently used in ERG recordings with corneal lens electrodes.

Reviewer #5 (Remarks to the Author):
I co-reviewed this manuscript with one of the reviewers who provided the listed reports. This is part of the Nature Communications initiative to facilitate training in peer review and to provide appropriate recognition for Early Career Researchers who co-review manuscripts.
RE: we thank the reviewer for their positive assessment.
